# Holistic Assessment of Rumen Microbiome Dynamics through Quantitative Metatranscriptomics Reveals Multifunctional Redundancy during Key Steps of Anaerobic Feed Degradation

**DOI:** 10.1128/mSystems.00038-18

**Published:** 2018-08-07

**Authors:** Andrea Söllinger, Alexander Tøsdal Tveit, Morten Poulsen, Samantha Joan Noel, Mia Bengtsson, Jörg Bernhardt, Anne Louise Frydendahl Hellwing, Peter Lund, Katharina Riedel, Christa Schleper, Ole Højberg, Tim Urich

**Affiliations:** a Department of Ecogenomics and Systems Biology, University of Vienna, Vienna, Austria; b Institute of Microbiology, University of Greifswald, Greifswald, Germany; c Department of Arctic and Marine Biology, the Arctic University of Norway, Tromsø, Norway; d Department of Animal Sciences, Aarhus University, Tjele, Denmark; Pacific Northwest National Laboratory

**Keywords:** archaea, *Methanomassiliicoccales*, carbohydrate active enzymes, metatranscriptomics, methane, methanogenesis, microbiome, rumen, volatile fatty acids

## Abstract

Ruminant livestock is a major source of the potent greenhouse gas methane. The complex rumen microbiome, consisting of bacteria, archaea, and microbial eukaryotes, facilitates anaerobic plant biomass degradation in the cow rumen, leading to methane emissions. Using an integrated approach combining multidomain quantitative metatranscriptomics with gas and volatile fatty acid (VFA) profiling, we aimed at obtaining the most comprehensive picture of the active rumen microbiome during feed degradation to date. Bacterial, archaeal, and eukaryotic biomass, but also methane emissions and VFA concentrations, increased drastically within an hour after feed intake. mRNA profiling revealed a dynamic response of carbohydrate-active enzyme transcripts, transcripts involved in VFA production and methanogenesis. While the relative abundances of functional transcripts did not mirror observed processes, such as methane emissions, transformation to mRNA abundance per gram of rumen fluid echoed ruminant processes. The microbiome composition was highly individual, with, e.g., ciliate, *Neocallimastigaceae*, *Prevotellaceae*, *Succinivibrionaceae*, and *Fibrobacteraceae* abundances differing between cows. Microbiome individuality was accompanied by inter- and intradomain multifunctional redundancy among microbiome members during feed degradation. This likely enabled the robust performance of the anaerobic degradation process in each rumen. *Neocallimastigaceae* and ciliates contributed an unexpectedly large share of transcripts for cellulose- and hemicellulose-degrading enzymes, respectively. Methyl-reducing but not CO_2_-reducing methanogens were positively correlated with methane emissions. While *Methanomassiliicoccales* switched from methanol to methylamines as electron acceptors, *Methanosphaera* became the dominating methanol-reducing methanogen. This study for the first time linked rumen meta-omics with processes and enabled holistic insights into the contribution of all microbiome members to feed degradation.

**IMPORTANCE** Ruminant animals, such as cows, live in a tight symbiotic association with microorganisms, allowing them to feed on otherwise indigestible plant biomass as food sources. Methane is produced as an end product of the anaerobic feed degradation in ruminants and is emitted to the atmosphere, making ruminant animals among the major anthropogenic sources of the potent greenhouse gas methane. Using newly developed quantitative metatranscriptomics for holistic microbiome analysis, we here identified bacterial, archaeal, and eukaryotic key players and the short-term dynamics of the rumen microbiome during anaerobic plant biomass degradation and subsequent methane emissions. These novel insights might pave the way for novel ecologically and economically sustainable methane mitigation strategies, much needed in times of global climate change.

**Author Video**: An author video summary of this article is available.

## Abstract (precis)

Ruminant animals, such as cows, live in a tight symbiotic association with microorganisms, allowing them to feed on otherwise indigestible plant biomass as food sources. Methane is produced as an end product of the anaerobic feed degradation in ruminants and is emitted to the atmosphere, making ruminant animals among the major anthropogenic sources of the potent greenhouse gas methane. Using newly developed quantitative metatranscriptomics for holistic microbiome analysis, we here identified bacterial, archaeal, and eukaryotic key players and the short-term dynamics of the rumen microbiome during anaerobic plant biomass degradation and subsequent methane emissions. These novel insights might pave the way for novel ecologically and economically sustainable methane mitigation strategies, much needed in times of global climate change.

## INTRODUCTION

Ruminant animals are the dominant large herbivores on Earth. Their evolutionary success is partly due to their tight symbiotic associations with commensal microorganisms that enable them to utilize otherwise indigestible plant biomass as food sources (1). Since their domestication in the Holocene, ruminants (in particular, cows) have provided humankind with various important goods. However, agricultural farming of cows is also a major source of the potent greenhouse gas (GHG) methane (CH_4_), having a global warming potential 34 times higher than carbon dioxide (2).

Cows possess a complex digestive system, including a four-compartment stomach, with the largest compartment being the rumen (3). The rumen is basically a big anaerobic fermentation chamber harboring the complex rumen microbiome (here defined as the entirety of all rumen microorganisms [i.e., the rumen microbiota] and their genetic repertoire) that catalyzes the anaerobic degradation of ingested plant biomass. During microbial hydrolysis and fermentation of plant fibers, volatile fatty acids (VFAs) are produced; the VFAs serve as the main energy source of the animal (4). A prominent end product of microbial degradation is CH_4_, produced by methanogenic archaea. Individual cows, or, more specifically, their symbiotic methanogens, produce up to 500 liters of CH_4_ per day (5), making ruminant livestock one of the major anthropogenic CH_4_ sources (6). Due to an increasing human world population, milk and meat demands are expected to double by 2050 (7), making the development of sustainable and productive animal farming systems a major challenge in agriculture (8). CH_4_ mitigation strategies are of not only ecological but also economic importance, as ruminant CH_4_ emissions represent an energy loss of 2% to 12% for the animal (5, 8).

Since the time of the pioneering work of Hungate and others (9–12), microbiologists have made large efforts to understand the structure-function relationships in the complex rumen microbiome, identifying the microorganisms that participate in certain steps of the anaerobic degradation pathway. More recently, the application of cultivation-independent molecular techniques has helped to uncover the high diversity of bacteria, archaea, and eukaryotes residing in the rumen and factors affecting community composition (see, e.g., reference 13). In addition, the usage of meta-omics techniques has paved the way for a better understanding of the rumen ecosystem and the microbial metabolic potential and activity in the rumen (reviewed in reference 14). These studies have revealed differences in rumen microbiome structure between animals emitting CH_4_ at low levels and those emitting it at high levels (see, e.g., references 15 and 16) and the effects of different diets on ruminant CH_4_ emissions (see, e.g., references 17, 18, and 19). New insights were also gained by identification of new members of functional groups, e.g., new fibrolytic and methanogenic community members (see, e.g., references 20, 21, 22, 23, and 24). Furthermore, the importance of diurnal microbiome dynamics for the understanding of VFA, H_2_, and CH_4_ production in the rumen was pointed out recently (25).

Despite these major advances, a holistic understanding of the rumen microbiome is still lacking, including answers to rather simple questions such as “who is doing what and when during feed degradation?” Such a fundamental understanding of the rumen ecosystem, as proposed by Hungate in the early 1960s (11), can help to specifically manipulate the rumen microbiome, to reduce CH_4_ emissions, without hampering animal productivity and milk and meat quality and without being harmful to the animal (14, 26).

To obtain a more comprehensive and holistic picture of the rumen microbiome activity during plant biomass degradation in lactating cows, we performed a short-term longitudinal study using an integrated approach, combining metatranscriptomics with gas and volatile fatty acid (VFA) profiling. By applying primer- and PCR-independent metatranscriptomics, we aimed at obtaining comprehensive multidomain profiles of the active rumen microbiome members (bacteria, eukaryotes, and archaea) and their functions in the key steps of anaerobic feed degradation, i.e., polysaccharide degradation, VFA production, and CH_4_ formation. We hypothesized that the microbiome exhibits a defined successional pattern, reflecting a cascade of hydrolytic, fermentative, and methanogenic steps, accompanied by distinct VFA and gas emission patterns. On the basis of a previous metatranscriptomic study from our laboratory (24) and work of others (see reference 27 and references therein), we hypothesized that the recently discovered *Methanomassiliicoccales* species are substantial contributors to ruminant CH_4_ emissions and would therefore show high activity after ruminant feed intake.

By transforming data representing the relative transcript abundances of rRNA and mRNA to abundance per gram of rumen fluid (quantitative metatranscriptomics), we were able to link rumen microorganisms and their transcription profiles to rumen processes, e.g., methane emission. Furthermore, we show extensive inter- and intradomain multifunctional redundancy among pro- and eukaryotic microbiome members at several key steps of the anaerobic degradation pathway.

## RESULTS

### Temporal dynamics of feed digestion.

**FIG 1:**
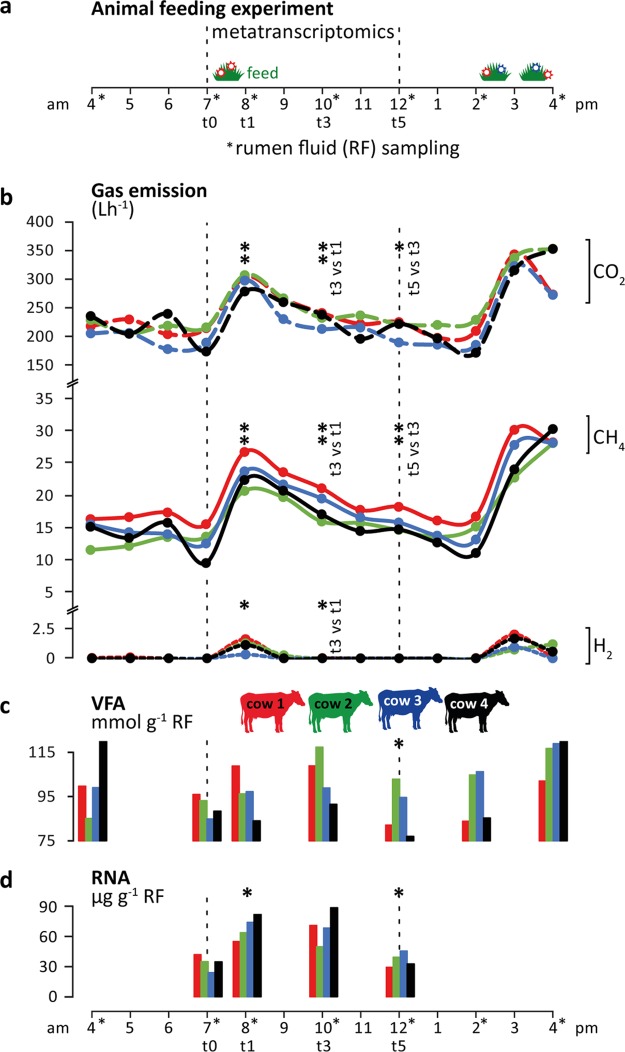
Ruminant gas emissions and volatile fatty acid (VFA) production. (a) Overview of the animal feeding trial during 12 h (4 a.m. to 4 p.m.) of sampling (for more details, see Materials and Methods). (b) Carbon dioxide (CO_2_), methane (CH_4_), and hydrogen (H_2_) emissions measured using open-circuit respiration chambers. (c and d) Total VFA concentrations (c) and total RNA content (d) quantified per gram of rumen fluid (RF). The color code indicates the four rumen-cannulated Holstein dairy cows. Bold asterisks indicate significant differences between the data for each respective time point and the previous one (*, *P* < 0.5; **, *P* < 0.01 [paired *t* test]).

To investigate the effect of feed intake on CH_4_ production by the rumen microbiome, we conducted a diurnal feeding experiment over 4 days. While rumen fluid samples were taken on day 2, we measured the CH_4_, CO_2_, and H_2_ emissions of four individual lactating Holstein cows on day 4 in open-circuit respiration chambers (Fig. 1; see also Table S1 and S2 in the supplemental material). Immediately after the morning feeding, CH_4_ and CO_2_ emissions significantly increased (mean increases, 1.9-fold and 1.5-fold, respectively), with all animals showing similar dynamics and magnitudes of gas production (Fig. 1b). The emissions dropped to before-feeding levels at 4 to 6 h after feed intake. H_2_ was detectable only during the first hour after feeding started (Fig. 1b), indicative of highly active H_2_-producing primary and secondary fermenters providing an excessive substrate for hydrogenotrophic methanogens. Similar dynamics in gas emissions were observed during afternoon feeding, with increasing gas emissions being measured immediately after the feeding started. However, as cows were fed *ad libitum* and took up feed continuously (feed was available between 2 p.m. and 4 a.m.), gas emissions stayed at high levels for several hours and eventually dropped at night.

10.1128/mSystems.00038-18.5 TABLE S1  Composition of the diet fed to the rumen-cannulated Holstein dairy cows. Cows were fed twice a day in a semirestrictive way (see Materials and Methods and Fig. 1a). Download TABLE S1, PDF file, 0.1 MB. Copyright © 2018 Söllinger et al. 2018 Söllinger et al. This content is distributed under the terms of the Creative Commons Attribution 4.0 International license.

10.1128/mSystems.00038-18.6 TABLE S2  Daily dry matter intake (in kilograms per day) for the four cows on the four experimental days. The dry matter intake was calculated as feed given minus leftovers multiplied by the dry matter concentration of the diet. The cows had dry matter intakes of 20.8 and 20.0 kg dry matter on the days with *ad libitum* and restricted feeding, respectively, and the intakes did not differ between the days with *ad libitum* feeding and restricted feeding (*t* test, *P* = 0.49). The cows had intakes of 14.5 ± 2.2 (mean ± standard deviation) and 9.8 ± 1.6 kg dry matter between 7 a.m. and 6 p.m. on days with *ad libitum* feeding (day 1 and day 3) and restricted feeding (day 2 and day 4), respectively, but the intakes did not differ between days 1 and 3 and days 2 and 4 (*t* test; day 1 versus day 3, *P* = 0.65; day 2 versus day 4, *P* = 0.95; *ad libitum* versus restricted, *P* = 0.0002). The cows had dry matter intakes of 2.7 ± 1.6 and 3.5 ± 0.6 kg between 7:00 a.m. and 8:00 a.m. on the days with rumen sampling and methane measurements, respectively, and the intakes did not differ between the days (*t* test, *P* = 0.43). For details on the experimental setup, see Materials and Methods. Download TABLE S2, PDF file, 0.1 MB. Copyright © 2018 Söllinger et al. 2018 Söllinger et al. This content is distributed under the terms of the Creative Commons Attribution 4.0 International license.

Likewise, the concentration of VFA in rumen fluid samples increased, with peak concentrations measured at 3 h and 2 h after the start of the morning and afternoon feedings, respectively (Fig. 1c) (Table 1), similarly to the results reported in reference 25. The VFA pools were more variable between the four cows than the gas emission profiles in terms of magnitude and temporal dynamics. The immediate accumulation of the fermentation products H_2_ and CO_2_ (i.e., substrates for methanogenesis) and of VFA after feeding indicated a fast physiological response of the rumen microbiome to feed intake, with enhanced fermentation rates leading to increased methanogenesis rates. Furthermore, a transient but significant increase in the RNA concentration in the rumen fluid was observed, which we consider a proxy for active microbiome biomass. Yields of total RNA per gram of rumen fluid were 34.1 ± 6.5, 69.2 ± 10.3, 70.0 ± 13.9, and 37.0 ± 6.3 µg at the time before feeding (t0) and at 1 h (t1), 3 h (t3), and 5 h (t5) after feeding started, respectively (Fig. 1d; see also Table S3). Rumen fluids for VFA quantification and RNA extraction were sampled prior to gas measurements, as it was not possible to perform sampling during respiration chamber measurements. The similar patterns in gas and VFA and RNA profiles reflected similar behaviors of the cows during the animal feeding trial (Table S2). Taken together, the patterns of the GHG emissions, VFA production, and RNA content indicated a consistent and fast growth response of the rumen microbiome and strong temporal dynamics on the process level (Fig. 1b to d; see also Fig. S1a in the supplemental material) within each individual cow.

10.1128/mSystems.00038-18.1 FIG S1  Biplots showing principal-component-analysis (PCA) results from rumen fluid metadata (a) and temporal separation and succession of rumen microbiomes (b). (a) The length of the arrows is equivalent to the contribution of the corresponding metadata to the ordination. Variables with a minor contribution to the ordination and VFAs of low abundance are not shown. The color code indicates the four different cows. RF, rumen fluid. (b) PCA of SSU rRNA transcript profiles. Triangles indicate taxa identified by indicator species analysis to be significantly higher in abundance at a given time (species scores were plotted onto the PCA biplot). Taxon numbering is as follows: 1, *Succinivibrionaceae*; 2, *Bacteroidaceae*; 3, *Synergistaceae*; 4, *Lactobacillaceae*; 5, *Burkholderiales*; 6, unassigned bacteria; 7, *Pseudomonadales*; 8, *Ascomycota*; 9, *Rhizobiales*; 10, *Eremoplastron*; 11, *Neocallimastigaceae*; 12, unassigned fungi; 13, unassigned *Tubulinea*; 14, *Rhizobiales*; 15, *Chytridiomycota*; 16, *Eccrinales*; 17, Viridiplantae; 18, *Victivallaceae*; 19, *Mastigamoeba*; 20, *Anaeroplasmataceae*; 21, candidate division TM6; 22, RFP12 gut group; 23, unassigned *Amoebozoa*; 24, unassigned *Spirotrichea*; 25, *Rhodospirillaceae*; 26, *Trichomonadidae*. Download FIG S1, PDF file, 1 MB. Copyright © 2018 Söllinger et al. 2018 Söllinger et al. This content is distributed under the terms of the Creative Commons Attribution 4.0 International license.

10.1128/mSystems.00038-18.7 TABLE S3  Overview of RNA extraction and sequencing and data processing results. Download TABLE S3, PDF file, 0.1 MB. Copyright © 2018 Söllinger et al. 2018 Söllinger et al. This content is distributed under the terms of the Creative Commons Attribution 4.0 International license.

**TABLE 1:** Volatile fatty acid (VFA) concentrations of all rumen fluid samples taken between 4 a.m. and 4 p.m.

Cow	Time	VFA concn (nmol·g^−1^ rumen fluid)												pH
		Formic acid	Acetic acid	Propionic acid	Iso- butyric acid	Butyric acid	Iso- valeric acid	Valeric acid	Caproic acid	Heptanoic acid	Lactic acid	Succinic acid	Total VFAs	
1	04 a.m.	0.0	63.4	16.8	0.8	16.2	0.5	1.8	0.2	0.0	0.0	0.0	99.7	
	07 a.m. (t0)	0.0	62.4	15.4	0.9	14.9	0.6	1.6	0.2	0.0	0.0	0.0	95.9	6.67
	08 a.m. (t1)	0.0	61.6	19.6	0.8	15.2	0.6	1.5	0.2	0.0	9.1	0.1	108.8	6.22
	10 a.m. (t3)	0.0	67.2	20.7	1.0	16.7	0.6	2.2	0.2	0.0	0.3	0.0	108.8	6.22
	12 a.m. (t5)	0.0	54.3	13.4	0.8	11.9	0.4	1.3	0.1	0.0	0.0	0.0	82.1	6.56
	02 p.m.	0.0	55.9	13.0	0.8	11.0	0.4	1.0	0.1	0.0	1.5	0.0	83.8	
	04 p.m.	0.0	59.3	22.6	0.8	15.9	0.7	1.7	0.2	0.0	0.9	0.0	102.1	
														
2	04 a.m.	0.0	49.7	23.3	0.8	7.3	0.4	3.0	0.5	0.1	0.0	0.0	85.1	
	07 a.m. (t0)	0.0	56.7	24.4	0.9	7.3	0.4	2.7	0.5	0.1	0.0	0.0	93.1	6.68
	08 a.m. (t1)	0.0	55.7	21.6	0.9	11.4	0.5	4.2	1.0	0.1	0.9	0.0	96.2	6.48
	10 a.m. (t3)	0.0	66.1	30.6	1.0	12.8	0.6	4.9	1.2	0.2	0.0	0.0	117.3	6.22
	12 a.m. (t5)	0.0	60.1	27.7	1.0	9.3	0.5	3.4	0.8	0.1	0.0	0.0	102.8	6.54
	02 p.m.	0.0	59.7	24.4	0.9	8.6	0.5	2.9	0.6	0.1	6.8	0.2	104.7	
	04 p.m.	0.3	54.2	23.0	0.7	15.3	0.5	4.9	1.4	0.1	16.2	0.2	116.7	
														
3	04 a.m.	0.0	59.9	24.8	0.7	10.6	0.4	2.1	0.5	0.1	0.0	0.0	99.0	
	07 a.m. (t0)	0.0	54.7	18.7	0.8	8.6	0.3	1.3	0.3	0.0	0.0	0.0	84.8	6.84
	08 a.m. (t1)	0.0	59.4	21.8	0.9	12.0	0.5	2.0	0.6	0.0	0.0	0.0	97.2	6.46
	10 a.m. (t3)	0.0	61.3	23.0	0.8	11.1	0.4	1.7	0.5	0.0	0.0	0.0	98.9	6.47
	12 a.m. (t5)	0.0	60.3	21.1	0.9	10.0	0.5	1.4	0.4	0.0	0.0	0.0	94.6	6.76
	02 p.m.	0.0	62.6	20.9	0.9	11.3	0.5	1.3	0.4	0.0	8.3	0.1	106.2	
	04 p.m.	0.0	65.5	29.1	1.0	17.6	0.8	2.4	0.8	0.0	1.7	0.0	118.9	
														
4	04 a.m.	0.0	70.2	32.3	0.8	12.4	0.5	3.2	0.4	0.0	0.0	0.0	119.9	
	07 a.m. (t0)	0.0	56.1	21.4	0.7	8.0	0.3	1.7	0.2	0.0	0.0	0.0	88.4	6.52
	08 a.m. (t1)	0.0	54.2	18.9	0.8	8.1	0.3	1.5	0.2	0.0	0.0	0.1	84.0	6.71
	10 a.m. (t3)	0.0	60.8	20.0	0.9	7.8	0.4	1.3	0.2	0.0	0.0	0.0	91.4	6.71
	12 a.m. (t5)	0.0	53.0	15.8	0.9	6.1	0.4	0.8	0.1	0.0	0.0	0.0	77.0	6.91
	02 p.m.	0.0	53.5	15.5	0.9	8.7	0.4	1.2	0.2	0.0	4.9	0.0	85.2	
	04 p.m.	0.0	64.5	24.8	0.9	21.6	0.7	3.5	0.6	0.0	10.7	0.0	127.3	

### Microbiome structure and dynamics.

**FIG 2:**
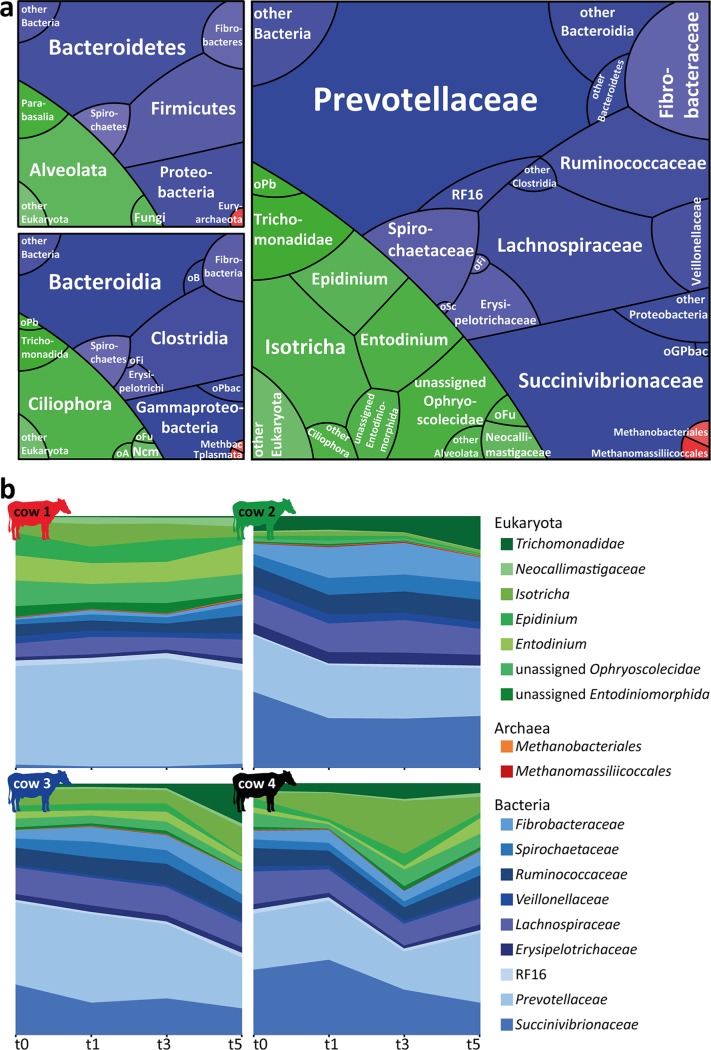
Rumen microbiome community composition and temporal dynamics. (a) Three domain profiles showing the overall rumen microbial community composition on the phylum (upper left panel), class (lower left panel), and family (right panel) levels. Tile sizes reflect the average relative abundances of eukaryotic (green), bacterial (blue), and archaeal (red) taxa observed in the 16 rumen metatranscriptomes. Taxa which could not be assigned on the family level and/or showed relative abundance levels of ≤1% are shown on higher taxonomic levels. oB, other *Bacteroidetes*; oPb, other *Parabasalia*; oFi, other *Firmicutes*; oPbac, other *Proteobacteria*; oFu, other fungi; oA, other *Alveolata*; Ncm, *Neocallimastigomycota*; Methbac, *Methanobacteria*; Tplasmata, *Thermoplasmata*; oSc, other *Spirochaetes*; oGPbac, other *Gammaproteobacteria*. (b) Microbial communities within each cow over time (low-abundance taxa were excluded). All taxa detected in the rumen microbiomes and their relative abundances are listed in Table S4.

We generated metatranscriptomes from the rumen fluid RNA using deep Illumina HiSeq paired-end sequencing and analyzed the rRNA and mRNA content (Fig. 1; see also Table S3). This primer- and PCR-independent approach enables the holistic detection and classification of eukarya, bacteria, and archaea, which is typically not possible via PCR/amplicon-based techniques (28, 29). The obtained three-domain profiles revealed that all major taxonomic groups known to occur in ruminants were present (Fig. 2a; see also Table S4), with eukaryotic, bacterial, and archaeal taxa accounting for 25.1% ± 10.5%, 74.5% ± 10.5%, and 0.3% ± 0.1% of the small-subunit (SSU) rRNA transcripts, respectively. Among the eukaryotes, ciliates were dominant, accounting for >70% of SSU rRNAs in 10 of 16 metatranscriptomes. The presence of *Entodinium* spp., *Epidinium* spp., and Eudiplodinium maggii and the absence of Polyplastron multivesiculatum were indicative of a type B ciliate community as typically found in cattle (30). Altogether, 155 different bacterial families were detected. Of these, transcripts attributable to 32 families were detected in all metatranscriptomes, with the most abundant (>1% of total rRNA reads) being *Prevotellaceae*, *Succinivibrionaceae*, *Lachnospiraceae*, *Ruminococcaceae*, *Fibrobacteraceae*, *Spirochaetaceae*, *Erysipelotrichaceae*, *Negativicutes* (formerly *Veillonellaceae*), and RF16. These nine taxa accounted on average for 92.3% of all bacterial SSU rRNA reads assigned to the family level (Fig. 2a), potentially representing the bovine core microbiome (13). All archaeal transcripts belonged to methanogens, with *Methanomassiliicoccales* and *Methanobacteriales* being the two dominant orders.

10.1128/mSystems.00038-18.8 TABLE S4  Relative percent abundances of eukaryotic, bacterial, and archaeal SSU rRNA reads. c1 to c4, cow 1 to 4. Download TABLE S4, PDF file, 0.3 MB. Copyright © 2018 Söllinger et al. 2018 Söllinger et al. This content is distributed under the terms of the Creative Commons Attribution 4.0 International license.

Although the same major eukaryotic, bacterial, and archaeal taxa were present in all microbiomes, the relative abundances of the microbiome members were highly individual in each cow (Fig. 2b; see also Table S4). For example, the proportions of eukaryotes in the individual rumen fluids ranged from 11.6% to 40.9% of SSU rRNA transcripts. The proportions of the two most prominent bacterial families, the *Prevotellaceae* and the *Succinivibrionaceae*, ranged from 21.0% to 66.0% and 1.6% to 34.4% of the microbiota, respectively. In contrast, the microbiota composition was remarkably stable in each cow rumen over the time course of the experiment (Fig. 2b) and did not show any consistent shifts in the individual cows, as revealed by several methods. Differential gene expression analysis of comparisons between sampling times showed no prokaryotic and no eukaryotic SSU rRNAs being differentially expressed at any time, except for three eukaryotic SSU rRNAs (i.e., *Epidinium*, *Eudiplodinium*, and unassigned *Litostomata*). The latter were significantly less abundant at t5 than at t3. Indicator species analysis identified several bacterial and eukaryotic taxa as significantly more abundant at certain time points (Fig. S1b). However, except for the *Trichomonadidae* (*Parabasalia*), a group of flagellated *Protozoa*, which were found to be significantly more abundant at t5, only low-abundance eukaryotic taxa were found to be indicators of the later time points. Furthermore, cow identity explained 64% of the variation in community composition (permutational multivariate analysis of variance [PERMANOVA] *P* = 0.001), while time did not explain a significant amount of variation (PERMANOVA *P* = 0.06).

**FIG 3:**
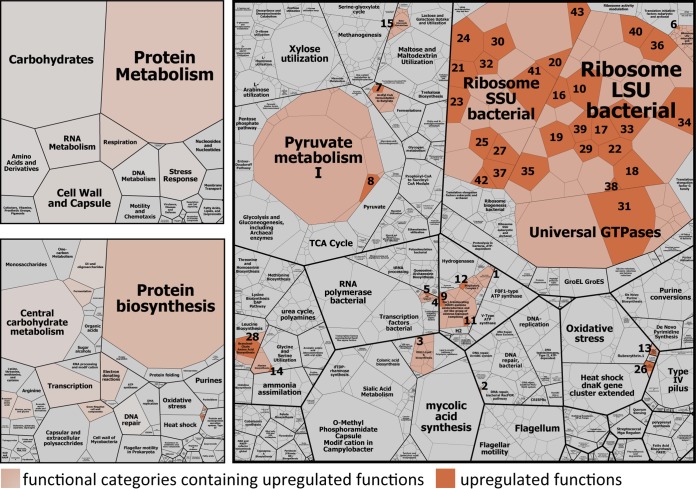
Global functional response of rumen microbiome to ruminant feed intake. Boxes show the mean relative abundances of all annotated mRNAs of eight rumen metatranscriptomes (t0 and t1 metatranscriptomes) in a hierarchical way. The color code indicates functional categories that were identified by differential gene expression analysis to be significantly higher expressed 1 h after the feeding (t1) than before the feeding (t0). The particular upregulated functions are colored in orange. All functions that were subjected to differential gene expression analysis are depicted (1,659 functions); low-abundance transcripts were excluded. For more details on the significantly higher expressed functions (e.g., functional assignment of the numbered tiles), see Table S5.

Analysis of mRNA gene expression profiles (based on all mRNA reads assigned to any SEED function) corroborated the notion that the observed process dynamics were an effect of an overall increase in activities rather than due to an induction of specific microbial taxa or metabolisms. In two time course transitions (t3 versus t1 and t5 versus t3), no significant differences were detected at all, while less than 3% of functional genes were significantly higher expressed 1 h after feeding (t1 versus t0). The majority (65%) fell into the subsystem protein biosynthesis (Fig. 3; see also Table S5), namely, transcripts of 12 SSU and 15 large-subunit (LSU) ribosomal proteins and the translation elongation factor G. Additionally, the relative abundances of two RNA polymerase subunit transcripts increased from t0 to t1. Only very few other transcripts, including transcripts involved in respiration, lipopolysaccharide biosynthesis (i.e., Kdo_2_-lipid A biosynthesis), alanine biosynthesis, biosynthesis of branched-chain amino acids, stress response, DNA repair, and VFA production/consumption, were significantly more abundant at t1 than at t0 (Fig. 3; see also Table S5). Our results suggest an immediate upregulation of the protein biosynthesis machinery as the major global response of the microbiome to feed intake.

10.1128/mSystems.00038-18.9 TABLE S5  Significantly higher expressed functions 1 h after ruminant feed intake. Download TABLE S5, PDF file, 0.2 MB. Copyright © 2018 Söllinger et al. 2018 Söllinger et al. This content is distributed under the terms of the Creative Commons Attribution 4.0 International license.

### Quantitative metatranscriptomics.

**FIG 4:**
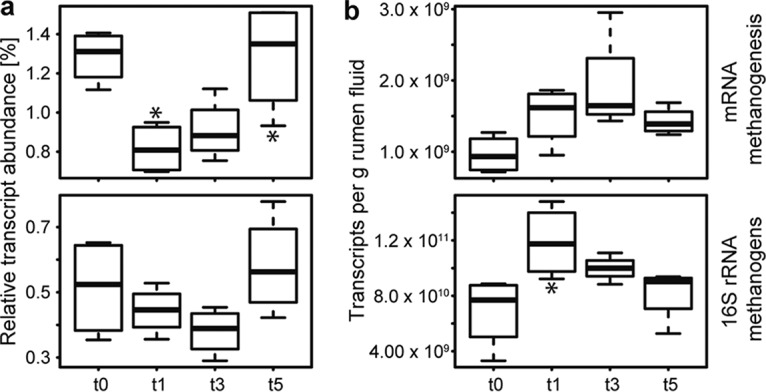
Comparison of relative and quantified transcript abundances of methanogens. Relative and quantified transcript abundances of methanogenesis-specific mRNA (upper box plots) and SSU rRNA of methanogens (lower box plots) are depicted in panels a and b, respectively. Data corresponding to mRNA reads assigned to the SEED subsystem methanogenesis and SSU rRNA reads assigned to methanogens are summarized. For details on the quantification, see Materials and Methods and equation 1. *x* axis: before feeding (t0) and 1 h (t1), 3 h (t3), and 5 h (t5) after the feeding started. Asterisks above and below the box plots indicate significant differences between the data for each respective time point and the previous one (*, *P* < 0.5; **, *P* < 0.01 [paired *t* test]).

We analyzed gene expression patterns of methanogens for successional changes during the experiment; such changes could explain the strong increase of CH_4_ emissions. However, the relative abundances of the methanogenesis-specific mRNAs and SSU rRNA transcripts of methanogen decreased at the time points with highest CH_4_ production (Fig. 4a). This pointed to a well-known problem in (meta-)omics approaches (31, 32), i.e., that of linking relative abundances of taxa or genes/transcripts with biogeochemical processes that are derived from heterogeneous data. We thus calculated transcript abundances per volume of rumen fluid (equation 1) by integrating relative transcript abundance data with total RNA concentrations extracted from rumen fluid. Using this quantitative metatranscriptomics approach, the transcript patterns of methanogens mirrored the observed dynamics in ruminant CH_4_ emissions, with an increase of transcripts per gram of rumen fluid at 1 and 3 h after the feeding started (t1 and t3) and a decrease 5 h after the feeding started (Fig. 4b). Similar effects were observed with gene expression patterns of other, high-level cellular functions, e.g., DNA replication.

### Major players in plant biomass degradation and CH_4_ production.

Using this quantitative approach, we conducted a broad, integrative mRNA screening to identify the major microbial players in three key steps of anaerobic plant biomass degradation: (i) breakdown of complex plant polysaccharides; (ii) carbohydrate fermentation to VFA; (iii) methanogenesis. We used rumen fluid as a proxy to analyze the complete anaerobic degradation cascade, although it has been shown that especially fibrolytic, particle-associated communities can differ (33, 34).

### Degradation of plant polysaccharides.

**FIG 5:**
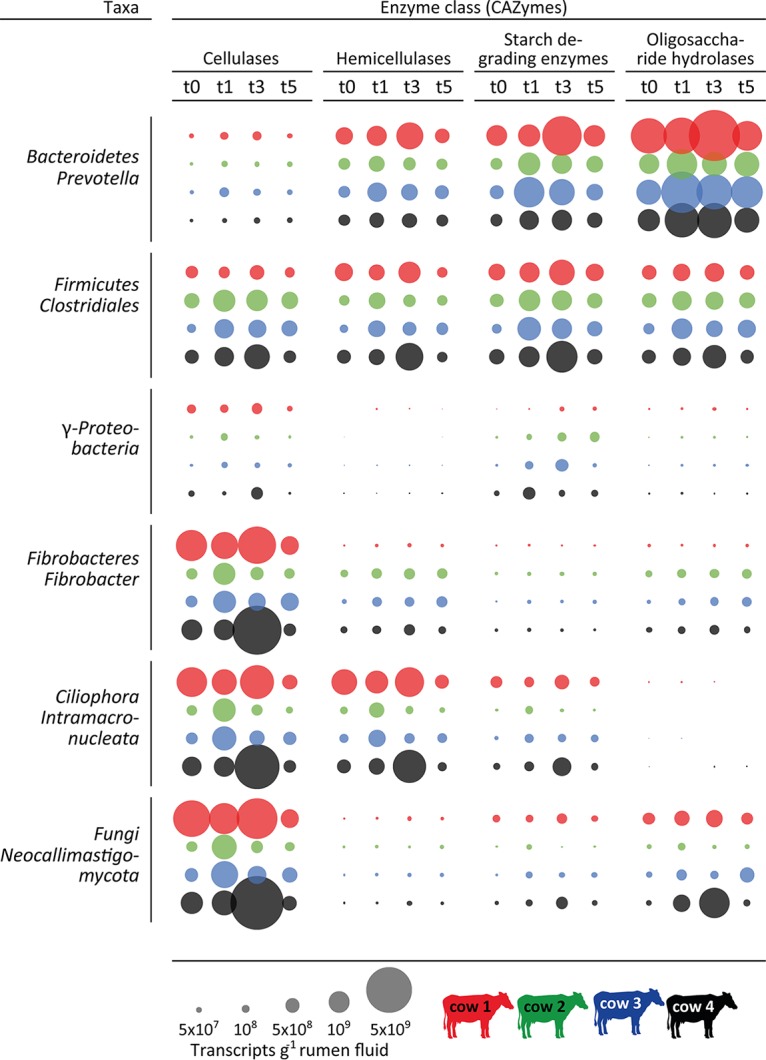
Dynamics and distribution of carbohydrate active enzymes (CAZymes) among the members of the rumen microbiome. Circles depict the quantified numbers of CAZyme transcripts (per gram of rumen fluid), summarized with respect to their activity (cellulases, hemicellulases, starch-degrading enzymes, and oligosaccharide hydrolases), separated for the major bacteria and eukarya involved in the breakdown of complex plant material (phylum level and dominant taxon within each phylum). The color code indicates the different cows and the different time points (gray scale of the columns): before feeding (t0) and 1 h (t1), 3 h (t3), and 5 h (t5) after the feeding started.

A screening for transcripts of carbohydrate active enzymes (CAZymes) revealed that the four dominant CAZyme categories were cellulases, hemicellulases, starch-degrading enzymes, and oligosaccharide hydrolases, accounting for 77.5% ± 2.1% of the total (see Fig. S2 and Table S6 in the supplemental material). We quantified and taxonomically classified these transcripts to reveal their distribution among the members of the rumen microbiome (Fig. 5). Three higher-level bacterial taxa and two eukaryotic taxa, namely, *Prevotellaceae* (*Bacteroidetes*), *Clostridiales* (*Firmicutes*), *Fibrobacter*, *Ciliophora*, and fungi (*Neocallimastigaceae*), were identified as predominantly involved. *Fibrobacteres* expressed the largest share of cellulase transcripts within the bacteria. Ciliates produced substantial amounts of hemicellulase and cellulase transcripts and surprisingly few transcripts encoding starch-degrading enzymes. Furthermore, anaerobic fungi of the *Neocallimastigaceae* produced the largest share of cellulase transcripts of all microorganisms. The abundant share of cellulase and hemicellulase transcripts encoded by *Clostridiales* establishes them as another key fiber-degrading bacterial group in the rumen (35). The data also show that *Prevotellaceae* primarily expressed genes encoding oligosaccharide hydrolases, starch-degrading enzymes, and hemicellulases but not cellulases. *Firmicutes* appeared to have the broadest capacity for polysaccharide degradation, with equal abundances of CAZyme transcripts in all four investigated categories. However, the *Firmicutes* (*Clostridiales*) comprised several different genera within the *Ruminococcaceae* and *Lachnospiraceae*, whereas *Fibrobacteres* and *Bacteroidetes* were each dominated by a single genus, *Fibrobacter* and *Prevotella*, respectively.

10.1128/mSystems.00038-18.2 FIG S2  Relative abundance of carbohydrate active enzymes (CAZymes) present in the cow rumen metatranscriptomes. CAZymes were summarized according to their substrate specificity/activity (see Table S6 for details on the underlying Pfam models). *x*-axis data represent the time before feeding (t0) and 1, 3, and 5 h after feeding started (t1, t3, and t5, respectively). Download FIG S2, PDF file, 0.4 MB. Copyright © 2018 Söllinger et al. 2018 Söllinger et al. This content is distributed under the terms of the Creative Commons Attribution 4.0 International license.

10.1128/mSystems.00038-18.10 TABLE S6  Raw count data of carbohydrate active enzymes detected in the rumen metatranscriptomes. c1 to c4, cow 1 to 4; t0, 7 h; t1, 8 h; t3, 10 h; t5, 12 h. Download TABLE S6, PDF file, 0.2 MB. Copyright © 2018 Söllinger et al. 2018 Söllinger et al. This content is distributed under the terms of the Creative Commons Attribution 4.0 International license.

The taxonomic distribution of CAZymes displayed strong differences between the cows, pointing to the same individuality as observed in the taxonomic composition of the rumen microbiome; e.g., eukaryotes dominated the cellulase transcript pools in cow 1 and cow 4, whereas *Fibrobacteres* and *Firmicutes* cellulase transcripts were as abundant as *Ciliophora* and *Fungi* cellulase transcripts in cow 2 and cow 3. Thus, expression of the different CAZyme categories by three to four different taxa shows a high functional redundancy for polysaccharide degradation in the rumen microbiome, within and between different domains of life.

### VFA production.

Acetate, propionate, and butyrate were the major VFAs, accounting for 60.4% ± 4.9%, 21.9% ± 3.4%, and 11.6% ± 2.7% of total VFAs, respectively. VFA concentrations in the rumen fluid increased after feed intake, while the pH dropped (Fig. 1c; see also Table 1). A strong negative linear correlation between pH and total VFA was observed between t0 and t5 [*r*_(14)_ = −0.84, *P* ≤ 0.0001]. Although no VFA production or absorption rates were measured, it has been shown that VFA concentrations are suitable proxies for production rates (4). Quantitative metatranscriptomics revealed the presence of transcripts for three complete acetate production pathways from pyruvate, i.e., directly (via pyruvate-ubiquinone oxidoreductase, *poxB*); via acetyl-coenzyme A (acetyl-CoA); and via acetyl-CoA and acetyl-P (Fig. S3a). The transcripts were assigned to *Bacteroidetes* (mainly *Prevotellaceae*) and *Firmicutes* (i.e., *Clostridiales* and *Negativicutes*), with transcript levels of *Prevotellaceae* exceeding those of *Firmicutes* by up to 30 times in the acetyl-CoA and acetyl-P pathways (Fig. S4a). In general, *poxB* transcript abundances (direct conversion of pyruvate to acetate) were 1 to 2 orders of magnitude lower than the abundances of the other pathways (Fig. S4a), with *Clostridiales poxB* transcripts dominating (by 1.4 to 64 times) over those of *Prevotellaceae poxB* in all samples. Together, these results suggest that *Prevotellaceae* species were the dominant acetate producers in this experiment.

10.1128/mSystems.00038-18.3 FIG S3  Dynamics and distribution of transcripts involved in acetate, propionate, and butyrate production in the rumen. (a) Heat map depicting the dynamics of quantified transcripts (indicated by their EC numbers) involved in acetate production (turnover) from pyruvate via acetyl-CoA, acetyl-CoA, and acetyl-P and direct production from pyruvate via *poxB* (pyruvate-ubiquinone oxidoreductase) by the rumen microbiome separated for the individual cows. ECs in bold indicate production-specific transcripts. The first row of the heat map depicts the acetate concentrations measured before feeding (t0) and 1 h (t1), 3 h (t3), and 5 h (t5) after the feeding started. Additionally, the acetate concentrations (indicated in nanomoles per gram of rumen fluid) for each cow are depicted above the heat map. Numbers with a superscript "1" symbol indicate the step of the pathway in which the respective enzymes are involved. (b) Heat map depicting the dynamics of quantified transcripts (indicated by their EC numbers) involved in propionate production (turnover) from succinate (succinate pathway) and lactate (acrylate pathway) and butyrate production (turnover) from acetyl-CoA by the rumen microbiome separated for the individual cows. ECs in bold indicate production-specific transcripts. Propionate and butyrate concentrations measured before feeding (t0) and 1 h (t1), 3 h (t3), and 5 h (t5) after the feeding started are depicted as heat maps and total concentrations (nanomoles per gram of rumen fluid). Numbers with a superscript "1" symbol indicate the step of the pathway in which the respective enzymes are involved. The butyryl-CoA–acetate CoA-transferase pathway (*Negativicutes*) was not detected in cow 1. Download FIG S3, PDF file, 0.7 MB. Copyright © 2018 Söllinger et al. 2018 Söllinger et al. This content is distributed under the terms of the Creative Commons Attribution 4.0 International license.

10.1128/mSystems.00038-18.4 FIG S4  Quantified transcripts involved in acetate, propionate, and butyrate production/turnover. (a) Bar charts showing quantified transcripts (per gram of rumen fluid) of *Bacteroidetes* (mainly *Prevotellaceae*), *Clostridiales* (*Clostridia*), and *Negativicutes* possibly involved in acetate production from pyruvate in the rumen, i.e., (A) production via acetyl-CoA, (B) production via acetyl-CoA and acetyl-P, and (C) direct production from pyruvate via *poxB* (pyruvate-ubiquinone oxidoreductase). Each bar summarizes all possibly involved enzymes (ECs) at a certain time point divided by the number of steps for each pathway (see Fig. S3). *x* axis: before feeding (t0) and 1 h (t1), 3 h (t3), and 5 h (t5) after feeding started. (b) Bar charts showing quantified transcripts (per gram of rumen fluid) of *Bacteroidetes* (mainly *Prevotellaceae*), *Clostridiales* (*Clostridia*), and *Negativicutes* possibly involved in propionate production (A) from succinate (succinate pathway) and lactate production (acrylate pathway) and butyrate production (B) from acetyl-CoA in the rumen. Each bar summarizes all possibly involved enzymes (ECs) at a certain time point divided by the number of steps for each pathway (see Fig. S3). *x* axis: before feeding (t0) and 1 h (t1), 3 h (t3), and 5 h (t5) after feeding started. Download FIG S4, PDF file, 0.5 MB. Copyright © 2018 Söllinger et al. 2018 Söllinger et al. This content is distributed under the terms of the Creative Commons Attribution 4.0 International license.

Transcript analysis revealed the presence of two distinct pathways for propionate production (Fig. S3b): (i) from succinate (succinate pathway) and (ii) from lactate (acrylate pathway). Transcript levels of *Prevotellaceae* again exceeded those of *Firmicutes* (i.e., *Clostridiales*) (by up to 20 times), suggesting that *Prevotellaceae* also dominated propionate production (Fig. S4b). Transcripts for two complete pathways possibly leading to butyrate production, i.e., the butyrate kinase pathway within *Clostridiales* and the butyryl-CoA–acetate CoA transferase pathway within *Negativicutes*, were detected within the *Firmicutes* (Fig. S3b and S4b). These pathways differ only in the last step, i.e., the conversion of butyryl-CoA to butyrate, which is performed in two steps via butyryl-P by *Clostridiales* and directly by *Negativicutes*. In general, transcript abundances of VFA production pathway enzymes mirrored the VFA concentration patterns, especially for acetate but, to a lesser extent, also for propionate and butyrate (Fig. S3a and b), with a peak in transcript abundance at t1 or t3 and a subsequent decrease in transcript abundance at t5. Again, the transcript abundances and their taxonomic distribution showed marked differences between the individual cows. For instance, the abundance of transcripts for acetate production via acetyl-CoA and propionate production assigned to *Bacteroidetes* (mainly *Prevotellaceae*) was much higher in cow 1 than in the other cows, reflecting the higher relative abundance of *Prevotellaceae* within cow 1. Furthermore, *Negativicutes* (formerly *Veillonellaceae*) had higher transcriptional activity for acetate production via acetyl-CoA and butyrate production than *Clostridiales* within cow 2 (Fig. S3a and b) but not within the other cows.

### Methanogenesis.

*Methanomassiliicoccales* and *Methanobacteriales* were the two dominant methanogenic orders, accounting for >99% of SSU rRNAs. All SSU rRNA transcripts assigned to the *Methanomassiliicoccales* belonged to the GIT clade (36), a sister lineage of *Methanomassiliicoccaceae*. Within the *Methanobacteriales*, the majority of the SSU rRNA transcripts belonged to the genus *Methanobrevibacter*, whereas *Methanosphaera* accounted for up to 13.3% (mean, 6.0%). Between 2.7% and 24.4% of *Methanobacteriales* SSU rRNA transcripts could not be assigned on the genus level (mean, 15.2%).

**FIG 6:**
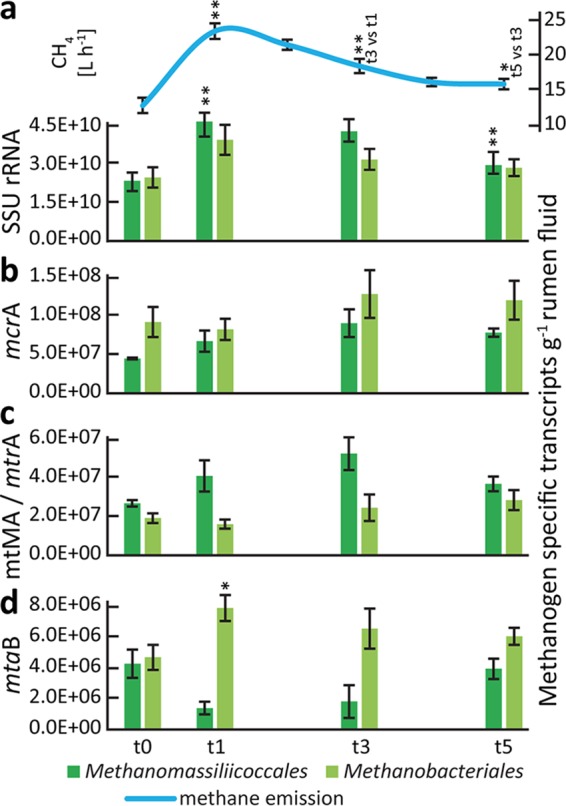
Methane and methanogen transcript dynamics during plant biomass degradation. (a) Methane emissions and quantified SSU rRNA transcripts of the two methanogen orders present in the rumen metatranscriptomes, *Methanomassiliicoccales* and *Methanobacteriales* (i.e., *Methanobrevibacter* and *Methanosphaera*), before feeding (t0) and 1 h (t1), 3 h (t3), and 5 h (t5) after the feeding started. (b) Quantification of transcripts of *mcrA* (functional marker for all methanogens). (c) Quantification of transcripts of *mtMA* (methylamine-specific methyltransferases) and *mtrA* (methyl-H_4_MPT:HS-CoM methyltransferase, alpha subunit), key transcripts in *Methanomassiliicoccales*- and *Methanobrevibacter*-specific methanogenesis, respectively. *mtMA* summarizes mono-, di-, and trimethylamine-specific methyltransferase (*mtmB*, *mtbB*, and *mttB*) transcripts, whereas *mttB* transcripts constitute >70% of the *mtMA* transcripts. (d) Quantified *mtaB* (methanol-specific methyltransferase) transcripts. *Methanomassiliicoccales* and *Methanosphaera mtaB* transcripts were negatively and positively correlated with CH_4_ emissions, respectively. Means of data determined from the four cows are shown for each time point; error bars depict standard errors of the means (SEM). Asterisks indicate significant differences between the data for each respective time point and the previous one (*, *P* < 0.5; **, *P* < 0.01).

The abundance of SSU rRNA transcripts of both groups followed the CH_4_ emission dynamics (Fig. 6a). However, only *Methanomassiliicoccales* SSU rRNA transcripts showed a strong positive linear correlation (*r*_*s*_ = 0.75, *P* < 0.001) and only their SSU rRNAs showed significant differences over time, similarly to the CH_4_ emissions (Fig. 6a). Methyl coenzyme M reductase (Mcr), the enzyme catalyzing the last step in methanogenesis, is conserved in all methanogenic archaea. The gene encoding the α-subunit of Mcr, *mcrA*, has thus been established as a functional and phylogenetic marker for methanogens (37, 38). No significant differences in *mcrA* transcript abundances were detected (Fig. 6b).

We searched for transcripts of key enzymes in taxon-specific methanogenesis pathways, including the following: (i) methylamine-specific methyltransferases (*mtMA*), involved in methanogenesis from methylamines by *Methanomassiliicoccales*; (ii) methyl-H_4_MPT:HS-CoM methyltransferase (*mtrA*), involved in methanogenesis from H_2_ and CO_2_ by *Methanobrevibacter*; and (iii) methanol-specific methyltransferase transcripts (*mtaB*), involved in methanogenesis from methanol by *Methanosphaera* and *Methanomassiliicoccales*. We observed the same pattern for *Methanomassiliicoccales mtMA* transcripts as for the SSU rRNA transcripts, i.e., a strong positive response to the feed intake (Fig. 6c). In contrast, no response of *Methanobrevibacter mtrA* transcript levels was observed. Immediately after feed intake, the abundance of *mtaB* transcripts of *Methanosphaera* increased, correlating positively with CH_4_ emissions (*r*_*s*_ = 0.59, *P* < 0.05) (Fig. 6d), while *Methanomassiliicoccales mtaB* transcripts negatively correlated with CH_4_ emissions (*r*_*s*_ = −0.63, *P* < 0.01). Taken together, these results indicate that only the methyl-reducing methanogens *Methanosphaera* and *Methanomassiliicoccales* responded to feed intake.

## DISCUSSION

In this study, we used an integrated approach, combining metatranscriptomics with targeted metabolomics (gas and VFA profiling) to holistically investigate temporal rumen microbiome dynamics during plant biomass degradation in lactating cows.

By integrating relative transcript abundances with RNA content per gram of rumen fluid, we were able to link rumen microorganisms and their activity to processes such as gas emissions and VFA production. Relative transcript abundances, which are commonly used in (meta-)transcriptomics, were not sufficient to establish this link (31, 32). Few studies have already applied quantitative metatranscriptomics; those that have done so predominantly examined marine ecosystems (see, e.g., references 39 and 40), focusing on bacteria and on nutrient cycling. Our study was the first host-associated quantitative metatranscriptomics study to link process data to microbiomes. Furthermore, our approach is different as we use total RNA concentrations instead of internal mRNA standards for “sizing up metatranscriptomics” (40). This quantitative approach allowed us to assess the contributions of major bacterial, eukaryotic, and archaeal taxa involved in the three key steps of anaerobic plant biomass degradation in the cow rumen. In fact, quantitative approaches in microbiome research have recently come to maturity (41).

By taxonomic classification of the small-subunit rRNA transcripts, we investigated if the rumen process dynamics (i.e., gas emissions and VFA production) were reflected in the composition of the microbiome. Our results showed that the microbiome composition was unexpectedly stable during feed digestion. The strong increase in the level of CH_4_ emissions after feeding was not related to a microbial community shift as we had hypothesized but to a fast growth response of the whole microbiome. This led to enhanced fermentation rates, reflected by the increase of CO_2_, H_2_, and VFA concentrations and an associated rise in methanogenesis rates. A similar dynamic of bacterial titers (number of SSU rRNA gene copies per milliliter of rumen fluid) as a response to feed intake was reported recently (25).

While they were stable over time, the individual microbiomes differed substantially between the four cows. Despite large differences in the abundances of the core bacterial and eukaryotic community members, these microbiomes exhibited similar fermentation characteristics, evidenced by gas and VFA patterns. This points toward extensive functional redundancy among rumen microbiome members, where multiple microorganisms possess the same functional trait(s) and can replace each other (42, 13).

Interdomain functional redundancy was widespread within the fibrolytic community, where eukaryotes and bacteria contributed various amounts of CAZyme transcripts within individual cows. For instance, most cellulase transcripts stemmed from two bacterial groups (*Fibrobacter* and *Clostridiales*) and two eukaryotic groups (*Neocallimastigaceae* and *Ciliophora*), with the eukaryotes producing the largest share of cellulase transcripts in two of the four cows. Interdomain functional redundancy was also observed within hemicellulose, starch, and oligosaccharide degradation, with marked differences between individual cows. Our results add to the growing notion that the eukaryotic contribution to fiber degradation has been underestimated in the past and support recent studies suggesting an important role of ciliates and fungi in ruminant (hemi)cellulose degradation (21, 43). Within the bacteria, *Bacteroidetes*, *Firmicutes*, and *Fibrobacteres* dominated the degradation of complex plant polysaccharides, with contributions of 48%, 31%, and 18% to the total CAZyme transcript pool. Similar observations were made in rumen metagenomic, metatranscriptomic, and metaproteomic studies (21, 44, 45). However, 10% of the CAZymes were assigned to *Proteobacteria* (40% *Bacteroidetes*, 30% *Firmicutes*) in the metagenomic analysis, and *Fibrobacteres* seemed to play a minor role (44). On the transcript level, Comtet-Marre et al. (21) also identified *Fibrobacteres*, in addition to *Bacteroidetes* and *Firmicutes*, as a bacterial contributor to cellulose, hemicellulose, and pectin degradation. In a recent metaproteomic study, more than two-thirds of all identified glycoside hydrolases were assigned to *Bacteroidetes*; *Firmicutes* and *Fibrobacteres* seem to play a minor role (45). However, other CAZyme categories were solely dominated by *Firmicutes*. Thus, the differences in the clustering of CAZymes in different categories can obstruct a direct comparison between studies.

Host individuality and functional redundancy were also revealed in the fermentation of carbohydrates to VFA. Three major, well-known VFA-producing taxa (46, 47) were identified, and their contribution to transcript pools of enzymes involved in VFA production was again found to be cow dependent. These taxa, *Bacteroidetes* (*Prevotellaceae*), *Clostridiales*, and *Negativicutes* (*Veillonellaceae*), produced acetate, propionate, and butyrate via different fermentative pathways; some were shared among taxa, and others were taxon specific. Although *Prevotellaceae* and *Clostridiales* in general dominated acetate/propionate and butyrate production, respectively, *Negativicutes* contributed substantially to butyrate production via the butyryl-CoA–acetate CoA-transferase pathway in cow 2 but not in the other three cows.

*Bacteroidetes* and *Firmicutes* were the two most abundant and active bacterial community members involved in the degradation of complex plant polysaccharides and the production of VFA. Notably, only one family and only one genus (i.e., *Prevotellaceae* and *Prevotella*) were dominant within the *Bacteroidetes*, while *Firmicutes* consisted of several families. This might explain why *Firmicutes* seemed to be more generalists than *Bacteroidetes*. *Firmicutes* species were involved in cellulose, hemicellulose, starch, and oligosaccharide degradation (with similar transcript abundances within these four categories), as well as in acetate, propionate, and butyrate production. In contrast, *Bacteroidetes* species were clearly dominant with respect to oligosaccharide hydrolysis and acetate and propionate production.

We also observed functional redundancy among the methanogens. The three detected groups (i.e., *Methanomassiliicoccales*, *Methanobrevibacter*, and *Methanosphaera*) differ fundamentally in their methanogenesis pathways. *Methanomassiliicoccales* species are H_2_-dependent methylotrophic methanogens, reducing methylamines and methanol to CH_4_ with H_2_ as an electron donor (48, 49). In contrast, *Methanobrevibacter* species produce CH_4_ mainly via the reduction of CO_2_ with H_2_ as an electron donor. *Methanosphaera* species, in turn, produce CH_4_ from methanol and H_2_ (50). The removal of H_2_ is important for the rumen ecosystem and for the host because low concentrations of H_2_ ensure high fermentation rates and efficient feed digestion (51). The longitudinal experimental setup revealed temporal dynamics in electron acceptor usage within the *Methanomassiliicoccales*, where the fraction of methanol-specific methyltransferase transcripts was much lower immediately after feeding, exhibiting an expression pattern opposite that seen with the methylamine-specific methyltransferases. In turn, it appeared that *Methanosphaera* dominated methanol reduction at these time points, showing once more the redundancy among organisms of the same functional guild. The root cause for this might be manifold, e.g., due to a higher substrate affinity of *Methanomassiliicoccales* for methylamines than for methanol or higher concentrations of methylamines. Alternatively, *Methanosphaera* could outcompete *Methanomassiliicoccales* for methanol under conditions of high H_2_ partial pressure. Taken together, the data suggest that methyl-reducing *Methanomassiliicoccales* species and, to a less extent, *Methanosphaera* species were responsible for the increase of CH_4_ emissions immediately after feed intake and not the CO_2_-reducing *Methanobrevibacter*. This is surprising, given that CO_2_ is a much more abundant methanogenesis substrate than methylamines and methanol. The sources of methylamines, i.e., glycine betaine (from beet), choline (from plant membranes), and methanol (from the hydrolysis of methanolic side groups in plant polysaccharides), are well known (52); however, the amounts of these substrates might vary substantially with different diets. Previous, less extensively temporally resolved work suggested that *Methanobrevibacter* was associated with high CH_4_ emissions (14, 53). However, a comparison of sheep rumen metagenomes and metatranscriptomes indicated that *Methanomassiliicoccales* are highly active community members in both high-level and low-level CH_4_ “emitters,” with abundances around 5 times higher in the metatranscriptomes than in the metagenomes (16). Furthermore, their transcript abundances were significantly higher in high-level CH_4_ emitters. Also, it was shown that *Methanomassiliicoccales* can represent the predominant active methanogens in cows (24). In fact, a need for more research on methyl-reducing methanogens in the rumen was pointed out recently (53), including quantifying their contribution to rumen methane production. Further studies on *Methanomassiliicoccales* and *Methanosphaera* physiology *in vitro* and metabolic interactions with the substrate-providing microorganisms *in situ* might identify novel targets for CH_4_ mitigation strategies, such as enzymes of the methyl-reducing pathway or the supply of methylated substrates. Such efforts might complement general methanogenesis inhibitors such as 3-nitrooxypropanol to achieve more-efficient methane mitigation (52).

To our knowledge, our report represents the first longitudinal integrated meta-omics analysis of the rumen microbiome during plant biomass degradation. It is another step toward a comprehensive system-level understanding of the dynamic rumen ecosystem, as already envisioned by Hungate and coworkers more than 50 years ago (11). Applying a quantitative metatranscriptomics approach, our study established a time-resolved link between microbiome structure and function and rumen processes. It revealed a rather simple response to feed intake, namely, a general growth of the whole community, without distinct successional stages during degradation. The individual cow microbiomes exhibited surprisingly high functional redundancy at several steps of the anaerobic degradation pathway, which can be seen as an example of the importance of multifunctional diversity for robustness of ecosystems, similarly to what has been found in terrestrial biomes (54). Our data furthermore point toward CH_4_ mitigation strategies that directly tackle the producers of CH_4_, since all other functional guilds show high organismic diversity, with individual taxa being replaceable by others.

## MATERIALS AND METHODS

### Animal feeding trial.

The animal feeding trial was conducted at the Department of Animal Sciences, Aarhus University (Denmark). The animal experiments were approved by the Experimental Animal Inspectorate under the Danish Ministry of Justice (journal number 2008/561-1500) (Fig. 1a). Four rumen-cannulated lactating Holstein dairy cows were fed a typical dairy cow diet containing mainly clover grass and corn silage (Table S1 and S2) twice a day in a semirestrictive way. The cows were in the second parity or later; they were 215 ± 112 (mean ± standard deviation) days in milk, had a live weight of 602 ± 20 kg, and had a milk yield of 33.5 ± 5.4 kg. Prior to the sampling, which was conducted over 4 days, the animals had been fed the corresponding diet continuously for more than 2 weeks. On day 1, cows were fed *ad libitum*. On day 2, the feed was removed at 4 a.m. and the cows were allowed to eat from 7 a.m. to 8 a.m. and again from 2 p.m. until 4 a.m. the next day. Rumen fluid was sampled at time points 4 a.m., 7 a.m., and 8 a.m. and every second hour until 10 p.m., with a final sampling performed at 4 a.m. on day 3. Rumen fluid was randomly sampled from different areas of the rumen, pooled, and filtered through sterile filter bags (Grade blender bags; VWR, Denmark) with a pore size of 0.5 mm. The pH of the rumen liquid samples was directly analyzed with a digital pH meter (Meterlab PHM 220; Radiometer, Denmark), and subsamples were frozen at −20°C for VFA analysis and other chemical analyses or were flash-frozen in liquid nitrogen and stored at −80°C for nucleic acid extraction. On day 3, animals were transferred to custom-built transparent polycarbonate open-circuit respiration chambers (1.45 by 3.90 by 2.45 m) and fed *ad libitum*. On day 4, the cows were fed as on day 2. CH_4_, CO_2_, and H_2_ were quantified continuously throughout the day.

### CH_4_, CO_2_, H_2_, and VFA quantification.

Open-circuit indirect-calorimetry-based respiration chambers (Columbus Instruments, Columbus, OH, USA), kept at slightly below ambient pressure, measured gas exchange (CH_4_, CO_2_, O_2_, and H_2_), airflow, and feed intake continuously during the experiment as described in detail in references 55 and 56. VFAs in the rumen liquid samples were quantified using a Hewlett-Packard gas chromatograph (model 6890; Agilent Technologies, Wilmington, DE) with a flame ionization detector and a 30-m SGE BP1 column (Scientific Instrument Services, Ringoes, NJ, USA) as described in reference 57.

### Nucleic acid extraction and linear RNA amplification.

Nucleic acids were extracted based on the methods described in references 58 and 28 (Fig. 1a). Extraction buffer and phenol/chloroform (Ambion) (5:1 [pH 4.5]; 0.5 ml of each) were added to a lysing matrix E tube (M.P. Biomedicals) containing approximately 0.25 g of rumen fluid sample. Cells were mechanically lysed using a FastPrep machine (M.P. Biomedicals) (speed, 5.5; 30 s) followed by nucleic acid precipitation with polyethylene glycol (PEG) 8000. All steps were performed on ice or at 4°C. Nucleic acids were resuspended in 50 µl diethyl pyrocarbonate (DEPC) H_2_O, and 1 µl of RNaseOUT (Thermo Fisher Scientific) was added. A 10-µl volume of nucleic acid extracts was subjected to DNase treatment (RQ1 DNase; Promega) and subsequent RNA purification (MEGAclear kit; Ambion). The quantity and quality of RNA were assessed via agarose gel electrophoresis and by the use of a NanoDrop spectrophotometer (ND-1000; Peqlab) and a Qubit assay kit (Thermo Fisher Scientific). The absence of DNA in the RNA preparations was verified by PCR assays targeting bacterial SSU rRNA genes and archaeal *mcrA* genes. The MessageAmp II bacterial kit (Ambion) was used according to the manufacturer’s manual to synthesize cDNA (via polyadenylation of template RNA and reverse transcription) and to perform *in vitro* transcription on the cDNA to amplify total RNA.

### Sequencing and sequence data preprocessing.

Illumina HiSeq 2500 paired-end (125-bp) sequencing was performed on cDNA at the Next Generation Sequencing Facility of the Vienna Biocenter Core Facilities. The template fragment size was adjusted such that paired sequence reads could be overlapped. We used PRINSEQ lite v. 0.20.4 (59) to apply quality filters and to trim the reads (parameters -min_len 180 -min_qual_mean 25 -ns_max_n 5 -trim_tail_right 15 -trim_tail_left 15). SortMeRNA v. 2.0 (60) was used to separate sequence reads into SSU rRNA, LSU rRNA, and putative mRNA reads. For more details and results of the initial data processing steps, see Table S3. All computations were performed using the CUBE computational resources, University of Vienna (Austria), or were run on the high-performance-computing (HPC) resource STALLO at the University of Tromsø (Norway).

### Metadata analysis.

The metadata (i.e., representing gas emissions [CH_4_, CO_2_, H_2_], feed and water intake, pH, volatile fatty acid [VFA] concentrations, and total RNA content) obtained from the rumen fluid samples used for metatranscriptomics (at t0, t1, t3, and t5) were subjected to principal-component analysis (PCA) using R (prcomp, ggbiplot). VFAs not detected in the majority of the samples were excluded from the principal-component analysis. Prior to the principal-component analysis, the dimensionally heterogeneous variables were standardized by applying z-scoring for each cow individually.

### Taxonomic classification of SSU rRNA reads.

We generated random SSU rRNA subsamples containing 50,000 reads of all SSU rRNA reads with a length of between 200 and 220 bp (45.8% ± 11.5% of total SSU rRNA reads). These subsamples were taxonomically classified with BLASTN against the SilvaMod rRNA reference database of CREST (Classification Resources for Environmental Sequence Tags) (61) and analyzed with MEGAN (62) v. 5.11.3 (parameters: minimum score 100, minimum support 1, top 2%, 50 best BLAST hits). Three domain profiles were visualized with tree maps based on CREST taxonomy. Rumen fluid microbial communities were subjected to various statistical analyses (i.e., principal-component analysis [PCA; function: rda], indicator species analysis [function: signassoc]), nonmetric multidimensional scaling (NMDS) (function: metaMDS) on a Bray-Curtis dissimilarity matrix (function: vegdist), permutational multivariate analysis of variance using distance matrices (PERMANOVA; function: Adonis), and differential gene expression analysis (function: glmFit) using R (63). The packages used were edgeR (64), vegan (65), indicspecies (66), and heat map3 (67). For the PCA and the NMDS, the taxon count matrices were normalized to the library sizes and transformed using separated z-scoring for the individual cows.

### Analysis of mRNA.

All putative mRNA reads were compared against the GenBank nr database using DIAMOND (68; v0.7.11, database as of December 2015; CUBE).

### CAZymes.

Randomly selected subsamples of 2 million nucleotide reads per data set were translated into open reading frames (ORFs) of 30 amino acids or longer. The ORFs were screened for protein families using HMMER and reference hidden Markov models (HMMER v3.0; screening against Pfam database v27) (69). All database hits with E values below a threshold of 10^−4^ were counted. Pfam annotations were screened for CAZymes using Pfam models of previously identified CAZymes (70) and additional rumen-relevant CAZymes (22) as well as CAZymes added to the Pfam-A database after the publications cited above and summarized into higher categories (Table S6). Translated reads assigned to any Pfam model of one of the four most dominant categories, i.e., cellulases, hemicellulases, starch-degrading enzymes, and oligosaccharide hydrolases (see Fig. S2 in the supplemental material), were extracted and blastp was used to obtain taxonomic information (blastp against the monthly updated nr database [04.2016]; CUBE). BLAST tables were imported into MEGAN (parameters: minimum score 50, minimum support 1, top 5%, 25 best blast hits) and further analyzed. CAZymes were quantified as described below (equation 1).

### VFA.

All mRNA reads assigned to any major taxa involved in the production of VFA, as identified by the SEED analyzer implemented in MEGAN, were subjected to further analysis to reconstruct major VFA production (turnover) pathways. These metatranscriptomic libraries were screened for all enzymes (via their respective EC numbers) involved in the production/turnover of acetate, propionate, and butyrate by blastp searches (E value threshold, 1e−10) using the metatranscriptomic libraries as queries against the UniRef50 database (monthly update of 12.2016; CUBE). The respective enzyme names were derived from the KEGG reference pathways and the literature (71, 72). Heat maps were constructed in R using quantified data (micrograms of transcripts per gram of rumen fluid; equation 1) and separated z-scoring for the individual cows.

### Methanogenesis.

Specific transcripts for methanogenesis were extracted from the DIAMOND annotation files via MEGAN and the implemented SEED analyzer. Assignments were critically manually evaluated; in cases of uncertainty, blastn was used to verify the accuracy and origin of the methanogenesis transcripts as well as of the SSU rRNA transcripts (against the NCBI and Silva databases as of September 2016). The transcripts were quantified (equation 1). Pearson’s product-moment correlations and Spearman rank correlation coefficients (rho = *r*_*s*_) between methanogen-specific transcripts (including pathway-specific key transcripts and SSU rRNA transcripts) were calculated, and paired *t* tests were used to assess temporal differences in transcript abundances (R functions: shapiro.test, cor.test, t.test).

### Differential gene expression analysis.

For mRNA analyses, DIAMOND blastx tables were imported into MEGAN (parameters: minimum score 40, minimum support 1, top 10%) and mRNA reads were functionally assigned using SEED, a curated categorization system where functional genes that are related to each other (e.g., by being part of the same biosynthesis pathway) are clustered together in a hierarchical way, via built-in mapping files. Relative abundances of mRNA reads assigned to a SEED function were subjected to differential gene expression analysis using edgeR (function: glmFit). Genes with low levels of expression were filtered out, and the default trimmed mean of M-values normalization (TMM) method was used to normalize the data. To account for the cow differences, a design matrix was constructed prior to the analysis to account for our experimental design and correct for batch effects (i.e., cow differences). Tree maps were created on the basis of the functional annotation using SEED, and the results of the differential gene expression analysis were mapped onto these tree maps. For rRNA analyses, taxon tables created as described above were subjected to differential gene expression analysis following the same workflow as that described for the SEED functions.

### Quantification of mRNA and rRNA transcripts per gram of rumen fluid.

We quantified mRNA and rRNA transcripts per gram of rumen fluid as follows:

(1) $$\text{transcript }A=\text{total RNA}\times \frac{x{\text{RNA}}_{r}}{x{\text{RNA}}_{r}+y{\text{RNA}}_{r}}\times \frac{\text{transcript }A_{r}}{x\text{RNA }{\text{subsample}}_{r}}\times \frac{N_{A}}{M\left(\text{Nt}\right)\times \text{transcript }A_{\text{length}}}$$

where total RNA is the amount of RNA (in micrograms) extracted per gram of rumen fluid; *x*RNA_*r*_, *y*RNA_*r*_, and *x*RNA subsample_*r*_ are the number of reads of mRNA or rRNA, rRNA or mRNA, and mRNA or rRNA subsamples used for functional annotation or taxonomic classification, respectively; transcript *A_r_* and transcript *A*_length_ are the number of reads assigned to a certain transcript and the length of the particular transcript, respectively; *N_A_* is the Avogadro constant; and *M*(Nt) is the average molecular weight of an single-stranded DNA (ssDNA) nucleotide (330 × 10^6^ µg·mol^−1^). For the transcript lengths, we used average values of 1,000 and 1,500/1,900 (prokaryotes/eukaryotes) nucleotides for mRNA and SSU rRNA transcripts, respectively. As previously observed (73), the polyadenylation that occurs during cDNA synthesis moderately enriches mRNA; therefore, a ratio of mRNA/total RNA reads of 1:25 was used to calculate transcript numbers per gram of rumen fluid, as this ratio was observed in a previous study on the rumen microbiome of cows of the same breed housed at the same facility and fed a diet containing similar amounts of neutral detergent fiber, crude protein, and fat (24).

### Accession number(s).

Raw sequence data have been submitted to the NCBI Sequence Read Archive (SRA) under accession numbers SAMN07313968, SAMN07313969, SAMN07313970, SAMN07313971, SAMN07313972, SAMN07313973, SAMN07313974, SAMN07313975, SAMN07313976, SAMN07313977, SAMN07313978, SAMN07313979, SAMN07313980, SAMN07313981, SAMN07313982, and SAMN07313983.
