# Supplementary material for: Holistic Assessment of Rumen Microbiome Dynamics through Quantitative Metatranscriptomics Reveals Multifunctional Redundancy during Key Steps of Anaerobic Feed Degradation
Source: mSystems. 2018 Aug 7;3(4):e00038-18. doi: 10.1128/mSystems.00038-18 (PMC6081794; doi:10.1128/mSystems.00038-18)
Supplement: TABLE S1 [file sys004182253st1.pdf]

Supplementary Table S1. Composition of the diet fed to the rumen-cannulated Holstein dairy cows. Cows were fed twice a day in a semi-restrictive way (see Methods section and Fig. 1a).

**Dietary composition**[a]	
Spring barley	217.9
Rape seed cake (12% fat)	221.5
Clover grass silage	303.5
Corn silage	244.8
Sodium Chloride	1.6
Vitamins and minerals	10.2
Chemical composition[1]	
Dry matter content [g/kg][b]	446
Ash[c]	65
Crude protein[d]	157
Fat[e]	36
Neutral detergent fibre[f]	340

[a] Given in g/kg DM if nothing else is stated. [b] The dry matter concentration was determined by drying the samples for 48 hours at 60°C. [c] Ash was determined by combustion at 525°C for 6 hours. [d] Nitrogen was determined by the Dumas principle as described by Hansen (1) using a Vario MAX CN (Elementar Analysesysteme GmbH, Hanau, Germany), and crude protein was calculated as N x 6.25. [e] Crude fat was measured by Soxhlet extraction with petroleum ether (Soltex 2050, Foss Analytical, Hillerød, Denmark) after hydrolysis with HCl (2). [f] Ash-free neutral detergent fibre was measured by FibertecTM M 6 system (Foss Analytical, Hillerød, Denmark) using heat stable amylase and sodium sulphite as described by Mertens (3).

References

1. Hansen B. 1989. Determination of nitrogen as elementary N, an alternative to Kjeldahl. Acta Agr Scand 39:113-118.
2. Stoldt W. 1952. Vorschlag zur Vereinheitlichung der Fettbestimmung in Lebensmitteln. Fette Seifen 54:206-207.
3. Mertens DR. 2002. Gravimetric determination of amylase-treated neutral detergent fiber in feeds with refluxing in beakers or crucibles: Collaborative study. J AOAC Int 85:1217-1240.
