# Supplementary material for: Holistic Assessment of Rumen Microbiome Dynamics through Quantitative Metatranscriptomics Reveals Multifunctional Redundancy during Key Steps of Anaerobic Feed Degradation
Source: mSystems. 2018 Aug 7;3(4):e00038-18. doi: 10.1128/mSystems.00038-18 (PMC6081794; doi:10.1128/mSystems.00038-18)
Supplement: TABLE S2 [file sys004182253st2.pdf]

Supplementary Table S2. Daily dry matter intake (kg/day) for the four cows on the four experimental days. The dry matter intake was calculated as feed given minus leftovers multiple with dry matter concentration of diet. The cows had a dry matter intake at 20.8 and 20.0 kg dry matter on the days with ad libitum and restricted feeding, respectively, and the intake did not differ between days with ad libitum and restricted feeding (t-test, P = 0.49). The cows had an intake of 14.5 ± 2.2 (mean ± standard deviation) and 9.8 ± 1.6 kg dry matter between 7 am and 6 pm on days with ad libitum feeding (day 1 and 3) and restricted feeding (day 2 and day 4), respectively, but the intake did not differ between day 1 and 3 and day 2 and 4 (t-test; day 1 versus day 3, P = 0.65; day 2 versus day 4, P = 0.95; ad libitum versus restricted, P = 0.0002). The cows had a dry matter intake at 2.7 ± 1.6 and 3.5 ± 0.6 kg between 7:00 and 8:00 on the days with rumen sampling and methane measurements, respectively, and the intake did not differ between the days (t-test, P = 0.43). For detail on the experimental setup see Methods section.

Time	Cow ID	Day 1	Day 2	Day 3	Day 4
24 h	Cow 1	19.2	15.4	19.9	20.1
	Cow 2	20.9	22.5	22.3	20.4
	Cow 3	19.4	18.2	19.1	20.1
	Cow 4	22.5	20.5	22.7	23.2
7 am – 6 pm	Cow 1	15.2	8.2	12.7	9.3
	Cow 2	18.2	12.1	17.3	10.4
	Cow 3	12.5	8.0	12.1	8.3
	Cow 4	14.0	10.7	14.5	11.4
7 am – 8 am	Cow 1		3.6		4.3
	Cow 2		4.4		3.3
	Cow 3		2.3		3.3
	Cow 4		0.6		3.0
