# Supplementary figures and images for: Holistic Assessment of Rumen Microbiome Dynamics through Quantitative Metatranscriptomics Reveals Multifunctional Redundancy during Key Steps of Anaerobic Feed Degradation
Source: mSystems. 2018 Aug 7;3(4):e00038-18. doi: 10.1128/mSystems.00038-18 (PMC6081794; doi:10.1128/mSystems.00038-18)

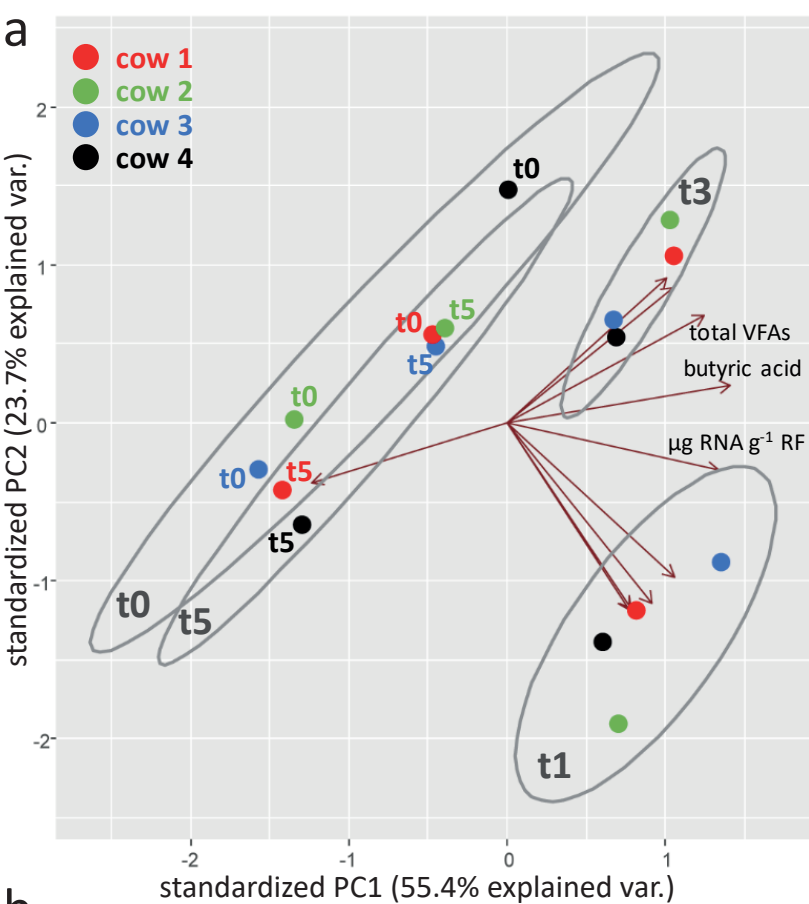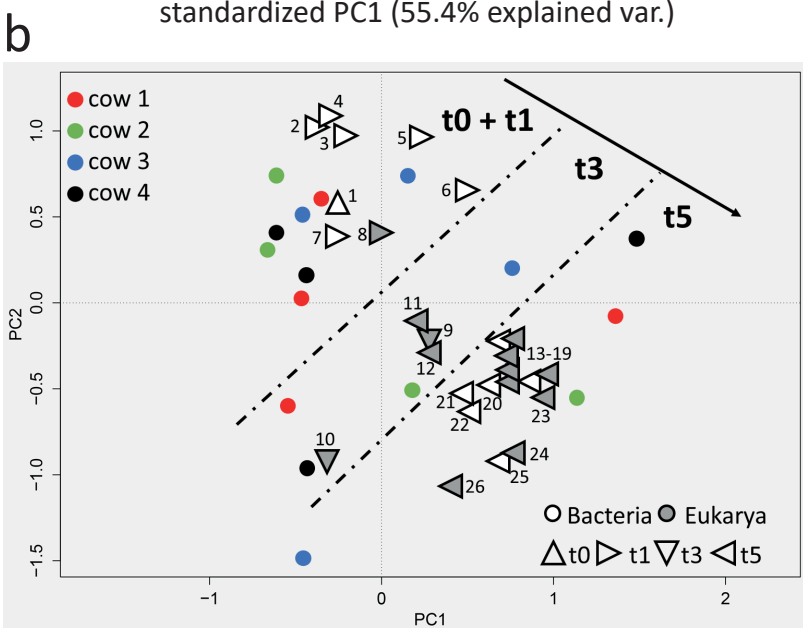

Supplement: FIG S1 [file sys004182253sf1.pdf]

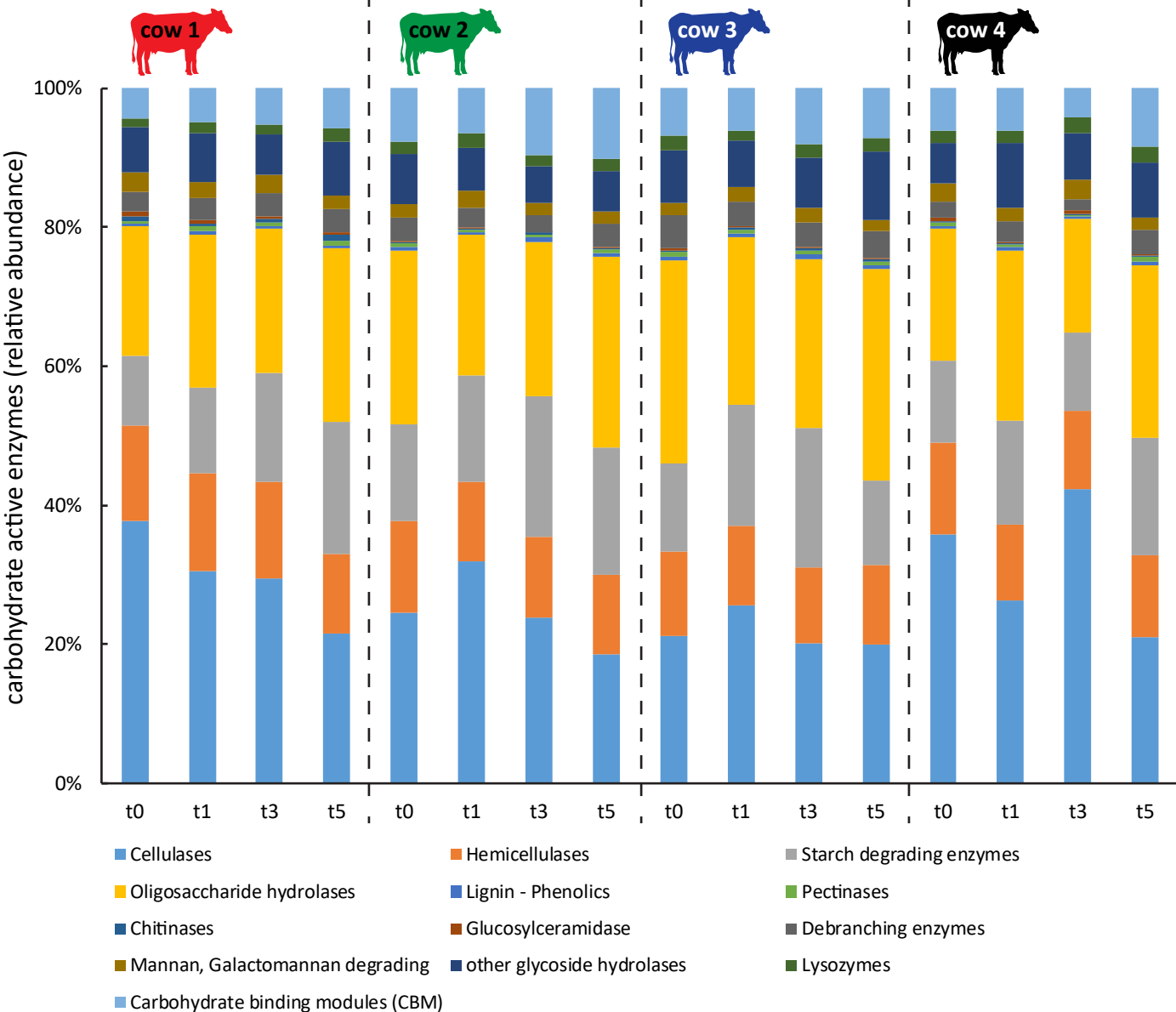

Supplement: FIG S2 [file sys004182253sf2.pdf]

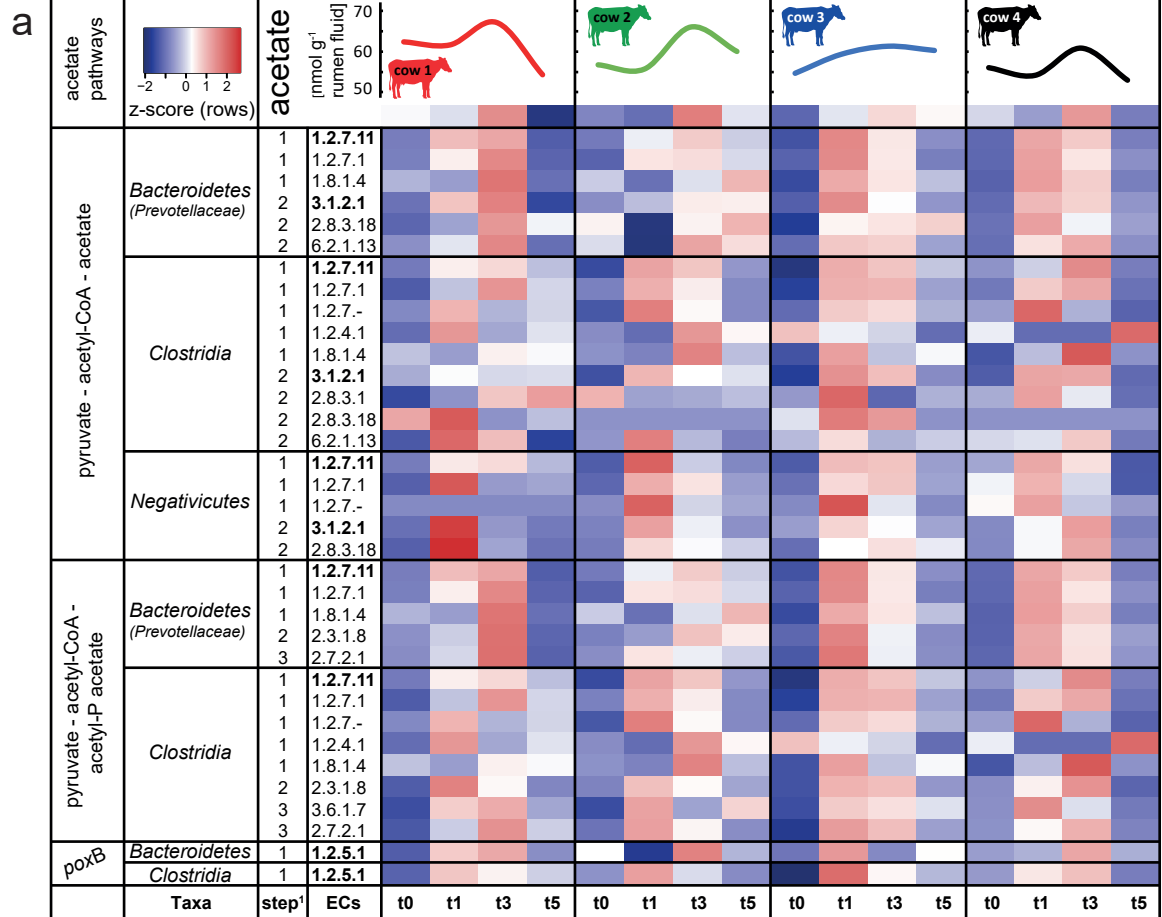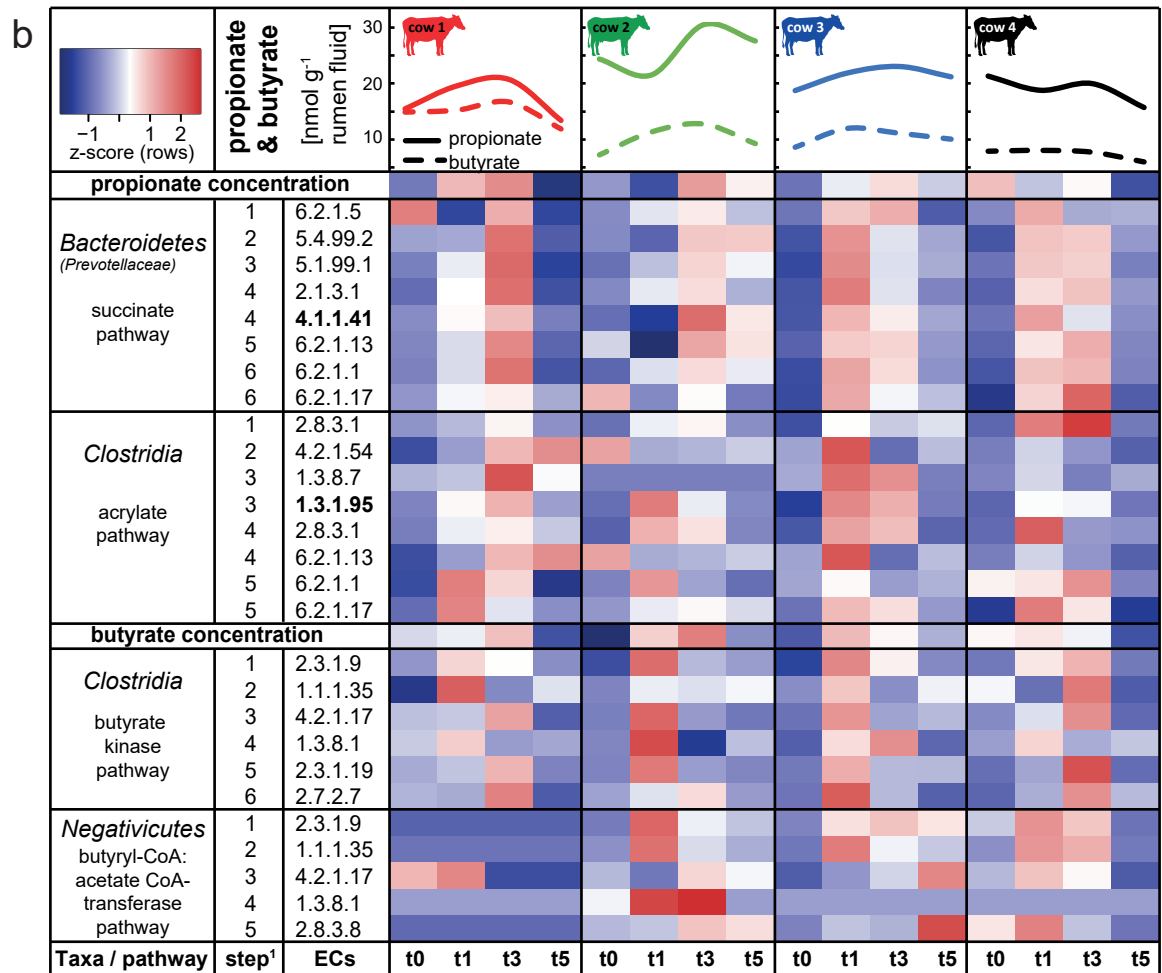

Supplement: FIG S3 [file sys004182253sf3.pdf]

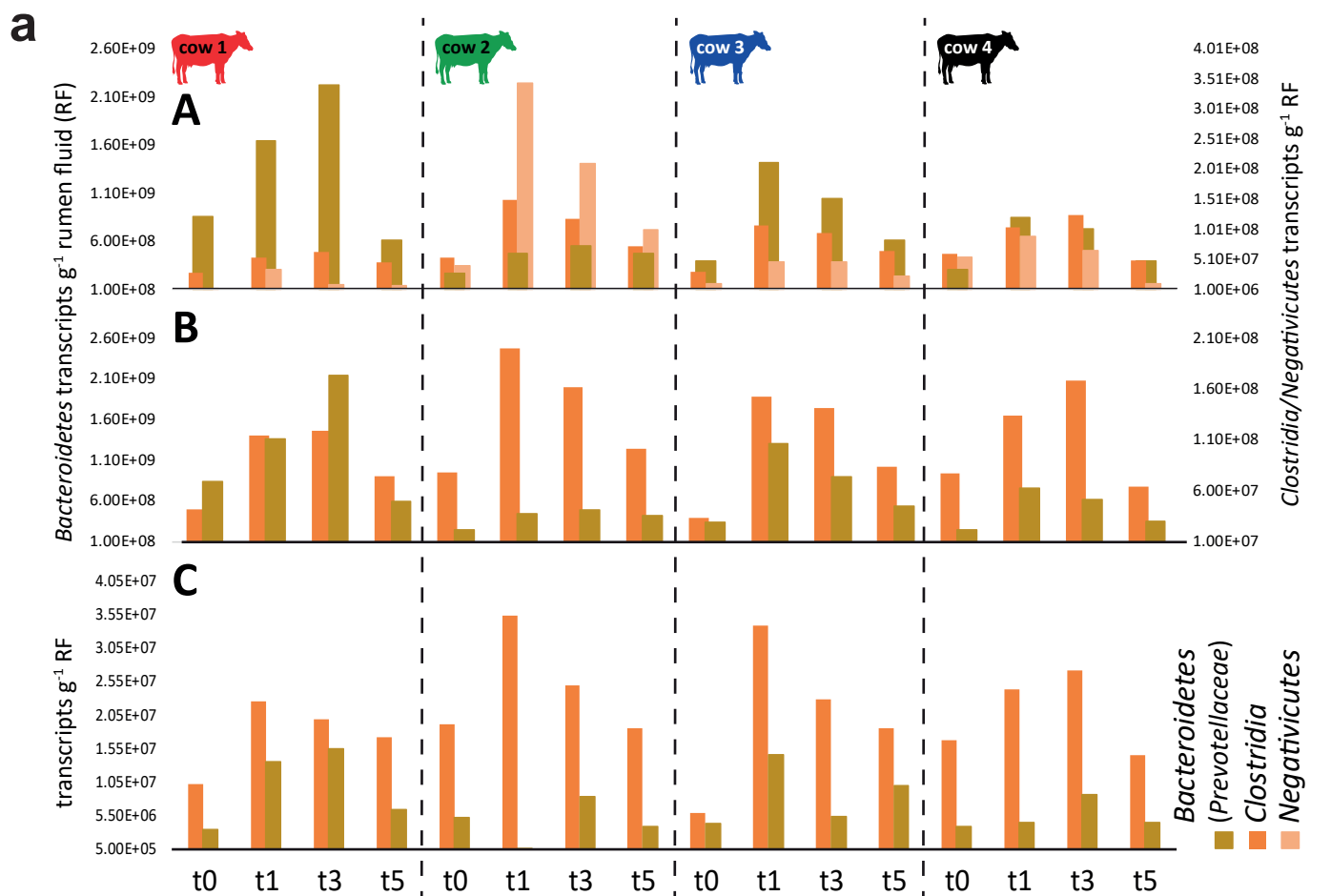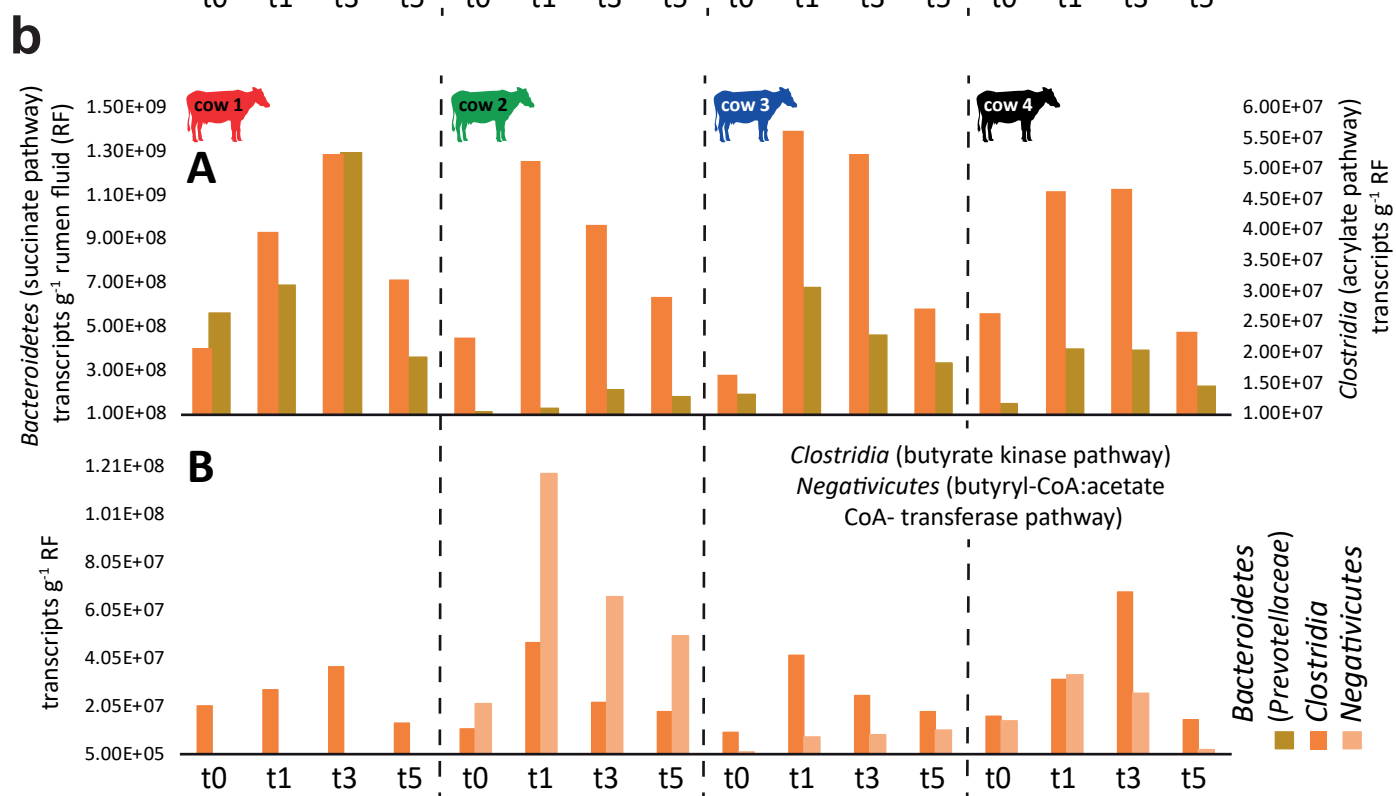

Supplement: FIG S4 [file sys004182253sf4.pdf]
