# Supplementary material for: Holistic Assessment of Rumen Microbiome Dynamics through Quantitative Metatranscriptomics Reveals Multifunctional Redundancy during Key Steps of Anaerobic Feed Degradation
Source: mSystems. 2018 Aug 7;3(4):e00038-18. doi: 10.1128/mSystems.00038-18 (PMC6081794; doi:10.1128/mSystems.00038-18)
Supplement: TABLE S3 [file sys004182253st3.pdf]

Supplementary Table S3. Overview of RNA extraction, sequencing and data processing results.

sample ID	µg RNA[a]	raw sequences[b]	after pre-processing[c]		SortMeRNA results[d]		annotation of mRNA	
		no. of read pairs	no. of reads	average read length	rRNA reads	mRNA reads	DIAMOND	Pfam[e]
c1_07h (t0)	42.3	65,317,599	60,466,622	214	52,334,976	8,131,646	3,365,969	410,419
c2_07h (t0)	35.1	59,186,503	51,812,887	214	42,488,514	9,324,373	3,365,969	290,817
c3_07h (t0)	24.1	75,330,840	53,433,658	215	43,639,108	9,794,550	3,365,969	368,829
c4_07h (t0)	35.0	53,259,101	62,901,199	217	52,540,980	10,360,219	3,365,969	342,208
c1_08h (t1)	55.3	66,549,413	59,656,114	209	51,068,193	8,587,921	3,365,969	491,989
c2_08h (t1)	64.3	67,146,045	59,149,252	215	50,666,579	8,482,673	3,365,969	424,483
c3_08h (t1)	74.6	63,379,266	53,417,765	198	44,106,590	9,311,175	3,365,969	417,605
c4_08h (t1)	82.6	63,533,899	36,342,383	191	29,672,369	6,670,014	3,365,969	304,716
c1_10h (t3)	71.5	64,634,701	58,801,041	219	50,430,178	8,370,863	3,365,969	542,244
c2_10h (t3)	50.2	66,938,760	42,031,443	223	35,888,902	6,142,541	3,365,969	399,755
c3_10h (t3)	68.9	67,312,364	58,695,012	207	48,385,273	10,309,739	3,365,969	384,594
c4_10h (t3)	89.3	65,740,801	48,605,118	217	42,611,059	5,994,059	3,365,969	478,864
c1_12h (t5)	29.5	62,897,615	58,136,430	224	52,364,413	5,772,017	3,365,969	467,908
c2_12h (t5)	39.6	66,954,185	62,273,447	214	54,042,405	8,231,042	3,365,969	387,441
c3_12h (t5)	45.9	71,966,923	50,534,927	221	44,197,682	6,337,245	3,365,969	370,793
c4_12h (t5)	32.8	62,238,171	57,829,771	220	49,765,881	8,063,890	3,365,969	395,907

[a]µg RNA extracted per gram rumen fluid [b] read length 125 bp; sequenced on a Illumina HiSeq 2500 125 bp; [c] after overlapping the reads, trimming and quality filtering; [d] SortMeRNA separated rRNA and putative mRNA reads based on the SILVA db; [e] number of reads annotated to a protein family from a subsample (2,000,000 reads) of total mRNA reads
