# Supplementary material for: Holistic Assessment of Rumen Microbiome Dynamics through Quantitative Metatranscriptomics Reveals Multifunctional Redundancy during Key Steps of Anaerobic Feed Degradation
Source: mSystems. 2018 Aug 7;3(4):e00038-18. doi: 10.1128/mSystems.00038-18 (PMC6081794; doi:10.1128/mSystems.00038-18)
Supplement: TABLE S4 [file sys004182253st4.pdf]

Supplementary Table S4. Relative abundance[a] of eukaryotic, bacterial and archaeal SSU rRNA reads [%]. c1 - c4, cow 1 – 4.

taxon string	c1_t0	c1_t1	c1_t3	c1_t5	c2_t0	c2_t1	c2_t3	c2_t5	c3_ t0	c3_t1	c3_t3	c3_t5	c4_ t0	c4_t1	c4_t3	c4_t5
unassigned Eukaryota	1.03	0.93	0.92	1.69	0.31	0.30	0.49	0.59	0.62	0.63	0.84	0.62	0.41	0.18	0.55	1.53
Metazoa	0.33	0.55	0.68	0.73	0.20	0.21	0.29	0.32	0.37	0.53	0.65	0.50	0.40	0.22	0.58	1.14
Alveolata; Apicomplexa; Aconoidasida; Piroplasmida	0.53	0.73	0.61	0.73	0.12	0.14	0.19	0.21	0.26	0.53	0.52	0.38	0.36	0.27	0.39	0.96
Alveolata; Ciliophora; Litostomatea; unassigned Litostomatea	0.12	0.14	0.19	0.18	0.02	0.02	0.03	0.01	0.06	0.06	0.11	0.05	0.07	0.06	0.14	0.12
Alveolata; Ciliophora; Litostomatea; other Litostomatea	0.03	0.06	0.04	0.04	0.00	0.00	0.00	0.00	0.02	0.01	0.02	0.02	0.01	0.01	0.04	0.02
Alveolata; Ciliophora; Litostomatea; unassigned Entodiniomorphida	3.85	2.42	3.83	1.70	0.43	0.67	0.34	0.17	0.96	0.94	1.17	0.32	1.10	0.16	1.49	1.01
Alveolata; Ciliophora; Litostomatea; unassigned Ophryoscolecidae	9.26	6.51	7.65	6.88	0.84	1.15	0.64	0.31	2.98	2.58	3.04	2.43	4.81	1.52	5.75	4.40
Alveolata; Ciliophora; Litostomatea; Entodinium	8.89	7.42	7.15	10.29	0.19	0.13	0.20	0.12	2.46	1.82	3.41	2.27	2.01	0.77	1.36	5.15
Alveolata; Ciliophora; Litostomatea; Epidinium	8.21	5.79	8.05	2.59	1.00	1.77	0.91	0.39	2.17	2.42	2.78	0.43	3.21	1.15	4.19	1.68
Alveolata; Ciliophora; Litostomatea; Eremoplastron	0.00	0.00	0.01	0.00	0.00	0.00	0.00	0.00	0.00	0.01	0.01	0.02	0.05	0.03	0.11	0.01
Alveolata; Ciliophora; Litostomatea; Eudiplodinium	0.00	0.00	0.01	0.00	0.00	0.00	0.00	0.00	0.00	0.01	0.01	0.00	0.02	0.02	0.09	0.01
Alveolata; Ciliophora; Litostomatea; Isotricha	4.07	8.04	5.71	3.50	1.19	1.73	1.01	0.95	5.39	4.95	4.33	9.79	2.03	9.16	18.69	5.32
Alveolata; Ciliophora; Litostomatea; Dasytricha	0.92	0.88	0.73	0.50	0.00	0.00	0.00	0.00	0.00	0.01	0.01	0.33	0.09	0.15	0.17	0.01
Alveolata; Ciliophora; Spirotrichea; unassigned Spirotrichea	0.05	0.07	0.07	0.11	0.03	0.04	0.04	0.06	0.09	0.10	0.14	0.18	0.12	0.05	0.16	0.19
Alveolata; Ciliophora; Colpodea; unassigned Colpodea	0.02	0.02	0.01	0.02	0.01	0.02	0.02	0.03	0.01	0.02	0.03	0.01	0.01	0.01	0.00	0.02
Alveolata; Ciliophora; Phyllopharyngea; Chonotrichia	0.14	0.13	0.13	0.11	0.03	0.05	0.03	0.04	0.23	0.32	0.18	0.09	0.12	0.06	0.13	0.52
Alveolata; low abundadnt Alveolata	0.05	0.04	0.05	0.10	0.05	0.03	0.02	0.03	0.04	0.04	0.05	0.02	0.03	0.02	0.04	0.05
Fungi; unassigned Fungi	0.03	0.04	0.06	0.06	0.01	0.00	0.00	0.01	0.02	0.02	0.01	0.02	0.02	0.00	0.01	0.02
Fungi; Dikarya; unassigned Dikarya	0.06	0.09	0.04	0.08	0.07	0.08	0.08	0.14	0.03	0.08	0.08	0.04	0.05	0.07	0.05	0.11
Fungi; Dikarya; Ascomycota	0.08	0.42	0.13	0.16	0.13	0.31	0.27	0.21	0.13	0.34	0.22	0.14	0.29	0.21	0.13	0.13
Fungi; Dikarya; Basidiomycota	0.05	0.05	0.03	0.07	0.03	0.03	0.02	0.05	0.11	0.09	0.08	0.07	0.08	0.03	0.07	0.18
Fungi; Chytridiomycota	0.02	0.03	0.02	0.04	0.02	0.01	0.01	0.03	0.01	0.01	0.01	0.02	0.02	0.01	0.01	0.03
Fungi; Neocallimastigomycota; Neocallimastigomycetes; Neocallimastigaceae	1.29	2.19	2.18	3.09	0.44	0.42	0.23	0.32	0.99	0.68	0.83	1.12	0.67	0.38	0.66	1.16
Fungi; low abundant Fungi	0.00	0.00	0.00	0.00	0.00	0.00	0.00	0.00	0.00	0.00	0.00	0.00	0.00	0.00	0.00	0.01
Parabasalia; unassigned Parabasalia	0.00	0.00	0.00	0.01	0.81	0.55	0.89	1.52	0.11	0.08	0.13	1.44	0.41	0.25	0.58	0.29
Parabasalia; Trichomonadida; Trichomonadidae	0.05	0.03	0.06	0.11	4.92	4.38	5.27	11.16	0.79	0.59	1.04	13.14	2.05	2.49	4.90	2.68
Parabasalia; Hypotrichomonadida	0.07	0.04	0.02	0.08	0.01	0.01	0.00	0.02	0.13	0.14	0.15	0.01	0.01	0.00	0.01	0.28
Parabasalia; low abundant Parabasalia	0.00	0.00	0.00	0.00	0.01	0.00	0.00	0.00	0.00	0.00	0.00	0.00	0.01	0.00	0.00	0.00
Stramenopiles	0.02	0.04	0.04	0.04	0.02	0.01	0.01	0.02	0.01	0.02	0.03	0.02	0.02	0.00	0.02	0.02
Viridiplantae	0.08	0.07	0.05	0.08	0.11	0.10	0.14	0.25	0.07	0.06	0.09	0.11	0.07	0.06	0.07	0.18
Rhizaria	0.08	0.07	0.12	0.12	0.03	0.02	0.03	0.04	0.09	0.14	0.29	0.11	0.06	0.04	0.10	0.30
Amoebozoa; unassigned Amoebozoa	0.01	0.01	0.01	0.02	0.02	0.02	0.02	0.04	0.01	0.01	0.02	0.02	0.00	0.01	0.02	0.04
Amoebozoa; Tubulinea; unassigned Tubulinea	0.02	0.02	0.02	0.02	0.02	0.01	0.03	0.04	0.01	0.01	0.02	0.01	0.01	0.02	0.01	0.03
Amoebozoa; Archamoebae; Mastigamoebidae; Mastigamoeba	0.50	0.33	0.39	0.64	0.47	0.48	0.61	0.81	0.26	0.38	0.39	0.37	0.24	0.14	0.36	0.84
Amoebozoa; Flabellinea; Vannellidae	0.08	0.07	0.07	0.13	0.01	0.01	0.02	0.02	0.03	0.02	0.07	0.03	0.01	0.03	0.02	0.08
Amoebozoa; low abundant Amoebozoa	0.00	0.00	0.01	0.01	0.01	0.02	0.01	0.01	0.01	0.00	0.00	0.01	0.01	0.00	0.01	0.00
Glaucocystophycea	0.04	0.04	0.04	0.06	0.01	0.01	0.00	0.00	0.00	0.01	0.01	0.00	0.00	0.00	0.00	0.01
Eccrinales	0.01	0.02	0.02	0.03	0.00	0.01	0.02	0.01	0.01	0.01	0.01	0.01	0.00	0.00	0.01	0.02
low abundant Eukaryota	0.03	0.03	0.04	0.08	0.02	0.02	0.02	0.04	0.02	0.03	0.04	0.02	0.01	0.00	0.02	0.09
unassigned Bacteria	0.04	0.09	0.04	0.11	0.10	0.12	0.11	0.09	0.16	0.22	0.20	0.16	0.11	0.17	0.12	0.18
Proteobacteria; unassigned Proteobacteria	0.00	0.02	0.02	0.03	0.01	0.08	0.24	0.16	0.02	0.21	0.35	0.01	0.05	0.29	0.04	0.08
Proteobacteria; Gammaproteobacteria; unassigned Gammaproteobacteria	0.00	0.02	0.01	0.02	0.03	0.05	0.12	0.04	0.01	0.05	0.07	0.01	0.03	0.03	0.02	0.02

Proteobacteria; Gammaproteobacteria; Pseudomonadales	0.01	0.05	0.01	0.02	0.01	0.02	0.01	0.01	0.00	0.01	0.00	0.01	0.02	0.02	0.00	0.01
Proteobacteria; Gammaproteobacteria; Aeromonadales; Succinivibrionaceae	1.56	0.97	0.96	1.73	27.84	18.39	17.91	18.53	18.09	11.34	12.80	9.53	23.46	27.10	16.41	10.93
Proteobacteria; Gammaproteobacteria; Cardiobacteriales	0.00	0.01	0.00	0.01	0.07	0.06	0.05	0.04	0.07	0.04	0.02	0.03	0.05	0.05	0.02	0.06
Proteobacteria; Alphaproteobacteria; unassigned Alphaproteobacteria	0.01	0.00	0.01	0.01	0.00	0.01	0.01	0.03	0.02	0.03	0.03	0.02	0.02	0.05	0.04	0.04
Proteobacteria; Alphaproteobacteria; Rhodospirillales; Rhodospirillaceae	0.03	0.03	0.03	0.06	0.24	0.27	0.29	0.46	0.91	0.96	1.10	2.14	1.22	1.25	1.79	1.77
Proteobacteria; Alphaproteobacteria; Rickettsiales	0.01	0.02	0.01	0.02	0.00	0.00	0.01	0.01	0.04	0.05	0.04	0.03	0.01	0.02	0.04	0.05
Proteobacteria; Alphaproteobacteria; Rhizobiales	0.00	0.01	0.01	0.01	0.01	0.01	0.01	0.01	0.00	0.01	0.01	0.03	0.01	0.01	0.01	0.03
Proteobacteria; Betaproteobacteria; Burkholderiales	0.02	0.04	0.01	0.01	0.03	0.03	0.01	0.02	0.00	0.03	0.02	0.00	0.01	0.01	0.01	0.01
Proteobacteria; Deltaproteobacteria; Desulfuromonadales	0.00	0.00	0.00	0.00	0.26	0.37	0.44	0.55	0.58	0.84	1.23	0.77	0.66	0.62	1.07	1.43
Proteobacteria; Deltaproteobacteria; Desulfobacterales	0.02	0.02	0.01	0.03	0.04	0.04	0.03	0.03	0.03	0.02	0.02	0.04	0.03	0.03	0.02	0.03
Proteobacteria; Deltaproteobacteria; Desulfovibrionales	0.02	0.06	0.02	0.05	0.06	0.06	0.03	0.03	0.03	0.05	0.04	0.03	0.04	0.03	0.03	0.02
Proteobacteria; low abundant Proteobacteria	0.02	0.08	0.02	0.04	0.05	0.04	0.06	0.07	0.07	0.07	0.06	0.07	0.04	0.06	0.06	0.07
Proteobacteria; Epsilonproteobacteria	0.00	0.00	0.00	0.00	0.03	0.01	0.01	0.02	0.02	0.01	0.01	0.01	0.01	0.02	0.01	0.02
Planctomycetes; Planctomycetacia; Planctomycetales; Planctomycetaceae	0.02	0.01	0.01	0.03	0.08	0.07	0.03	0.07	0.07	0.07	0.06	0.06	0.06	0.05	0.03	0.06
Planctomycetes; low abundant Planctomycetes	0.00	0.00	0.00	0.00	0.00	0.00	0.00	0.00	0.00	0.00	0.00	0.00	0.00	0.00	0.00	0.00
Cyanobacteria; Melainabacteria	0.18	0.21	0.21	0.15	0.14	0.21	0.17	0.20	0.26	0.38	0.43	0.56	0.35	0.54	0.48	0.34
Bacteroidetes; unassigned Bacteroidetes	0.03	0.03	0.03	0.04	0.01	0.01	0.01	0.02	0.04	0.04	0.05	0.03	0.02	0.02	0.03	0.07
Bacteroidetes; Flavobacteria; Flavobacteriales	0.01	0.03	0.01	0.01	0.01	0.01	0.00	0.01	0.01	0.02	0.02	0.00	0.00	0.01	0.01	0.02
Bacteroidetes; Sphingobacteria; Sphingobacteriales	0.01	0.02	0.01	0.03	0.01	0.01	0.01	0.00	0.03	0.03	0.01	0.02	0.01	0.01	0.02	0.03
Bacteroidetes; Bacteroidia; Bacteroidales; S24-7	0.28	0.47	0.31	0.48	0.61	0.60	0.66	0.91	0.49	0.71	0.75	0.28	0.40	0.42	0.27	0.49
Bacteroidetes; Bacteroidia; Bacteroidales; Bacteroidaceae	0.09	0.21	0.11	0.10	0.06	0.06	0.05	0.06	0.11	0.12	0.09	0.07	0.07	0.09	0.06	0.06
Bacteroidetes; Bacteroidia; Bacteroidales; Prevotellaceae	35.31	36.53	39.02	32.44	20.17	19.11	18.33	16.61	28.81	30.24	25.04	17.32	19.91	20.85	13.35	22.70
Bacteroidetes; Bacteroidia; Bacteroidales; RF16	2.09	1.92	1.87	2.34	0.67	0.64	1.16	0.93	0.91	0.84	0.94	1.63	1.48	1.19	1.10	0.99
Bacteroidetes; Bacteroidia; Bacteroidales; Rikenellaceae	1.29	1.25	0.93	1.03	0.91	0.72	0.57	0.67	1.02	1.09	0.91	0.62	0.78	0.90	0.48	0.74
Bacteroidetes; Bacteroidia; Bacteroidales; Porphyromonadaceae	0.03	0.03	0.02	0.05	0.09	0.16	0.17	0.24	0.04	0.08	0.08	0.03	0.03	0.02	0.03	0.05
Bacteroidetes; Bacteroidia; Bacteroidales; BS11 gut group	0.89	1.00	0.75	0.77	0.74	0.69	0.70	0.74	0.80	1.02	0.95	0.65	0.78	1.17	0.54	0.71
Bacteroidetes; Bacteroidia; Bacteroidales; other Bacteroidales families	0.02	0.02	0.01	0.02	0.08	0.06	0.02	0.03	0.03	0.03	0.03	0.01	0.02	0.03	0.01	0.02
Bacteroidetes; Cytophagia; Cytophagales	0.05	0.06	0.06	0.06	0.01	0.00	0.00	0.01	0.05	0.07	0.07	0.07	0.04	0.05	0.06	0.10
Bacteroidetes; low abundant Bacteroidetes	0.84	0.76	0.68	0.86	0.43	0.45	0.49	0.54	0.97	1.05	0.95	0.61	0.74	0.87	0.63	0.75
Actinobacteria; Actinobacteria; Corynebacteriales	0.02	0.06	0.03	0.03	0.08	0.05	0.02	0.04	0.15	0.07	0.01	0.01	0.02	0.03	0.01	0.02
Actinobacteria; Actinobacteria; Bifidobacteriales; Bifidobacteriaceae	0.03	0.02	0.01	0.02	0.09	0.10	0.08	0.08	0.37	0.34	0.38	0.02	0.05	0.07	0.03	0.20
Actinobacteria; Coriobacteriia; Coriobacteriales; Coriobacteriaceae	0.12	0.31	0.12	0.20	0.12	0.11	0.09	0.08	0.17	0.18	0.18	0.12	0.15	0.13	0.06	0.12
Actinobacteria; low abundant Actinobacteria	0.01	0.02	0.01	0.01	0.01	0.01	0.01	0.01	0.00	0.01	0.02	0.01	0.01	0.01	0.00	0.01
Firmicutes; unassigned Firmicutes	0.00	0.01	0.00	0.00	0.03	0.02	0.03	0.01	0.01	0.01	0.01	0.01	0.01	0.02	0.01	0.00
Firmicutes; Bacilli; Lactobacillales; Streptococcaceae	0.00	0.00	0.00	0.00	0.10	0.05	0.05	0.06	0.14	0.12	0.14	0.01	0.01	0.01	0.01	0.12
Firmicutes; Bacilli; Lactobacillales; Lactobacillaceae	0.01	0.03	0.01	0.01	0.03	0.04	0.02	0.03	0.03	0.04	0.03	0.02	0.03	0.02	0.01	0.01
Firmicutes; Erysipelotrichi; Erysipelotrichales; Erysipelotrichaceae	1.30	1.22	1.17	2.08	4.08	4.05	4.22	3.78	1.98	2.15	2.08	1.96	1.90	1.62	1.05	1.94
Firmicutes; Clostridia; Clostridiales; unassigned Clostridiales	0.06	0.07	0.06	0.14	0.11	0.09	0.09	0.10	0.10	0.10	0.12	0.09	0.11	0.11	0.07	0.14
Firmicutes; Clostridia; Clostridiales; Lachnospiraceae	4.85	6.09	4.43	6.49	10.30	10.40	11.62	9.12	9.30	9.46	9.48	8.34	11.47	8.28	7.71	8.48
Firmicutes; Clostridia; Clostridiales; Veillonellaceae	2.38	2.36	1.81	2.11	3.10	3.27	2.69	2.44	1.02	1.01	0.81	1.16	1.83	1.44	1.05	0.72
Firmicutes; Clostridia; Clostridiales; Ruminococcaceae	4.49	3.55	3.28	6.16	7.06	6.94	7.55	7.76	6.00	6.08	7.13	5.50	6.51	5.19	4.80	6.81
Firmicutes; Clostridia; Clostridiales; Family XIII Incertae Sedis	0.17	0.14	0.15	0.32	0.30	0.24	0.25	0.22	0.31	0.28	0.25	0.18	0.22	0.22	0.15	0.25
Firmicutes; Clostridia; Clostridiales; Eubacteriaceae	0.03	0.02	0.03	0.03	0.46	0.34	0.32	0.31	0.03	0.02	0.02	0.05	0.05	0.04	0.04	0.05
Firmicutes; low abundant Firmicutes	0.01	0.03	0.02	0.01	0.03	0.03	0.01	0.02	0.02	0.03	0.04	0.01	0.01	0.03	0.02	0.04
Verrucomicrobia; Opitutae	0.00	0.00	0.00	0.00	0.02	0.03	0.03	0.03	0.11	0.16	0.14	0.10	0.03	0.08	0.04	0.16
Verrucomicrobia; low abundant Verrucomicrobia	0.00	0.00	0.00	0.00	0.00	0.00	0.00	0.00	0.00	0.00	0.00	0.00	0.00	0.00	0.00	0.00

Tenericutes; Mollicutes; Anaeroplasmatales; Anaeroplasmataceae	0.60	0.43	0.61	1.16	0.18	0.22	0.29	0.28	0.52	0.50	0.45	0.53	0.35	0.25	0.24	0.56
Tenericutes; Mollicutes; other Mollicutes	0.02	0.03	0.03	0.11	0.28	0.21	0.23	0.21	0.28	0.25	0.26	0.36	0.45	0.28	0.29	0.32
Tenericutes; low abundant Tenericutes	0.02	0.02	0.01	0.03	0.01	0.01	0.01	0.03	0.02	0.02	0.03	0.01	0.02	0.01	0.01	0.03
RF3	0.04	0.02	0.02	0.06	0.01	0.00	0.02	0.02	0.04	0.04	0.03	0.02	0.02	0.00	0.00	0.03
Lentisphaerae; Lentisphaeria; Victivallales; Victivallaceae	0.01	0.02	0.01	0.03	0.13	0.18	0.18	0.26	0.13	0.16	0.18	0.23	0.08	0.09	0.07	0.18
Lentisphaerae; Lentisphaeria; RFP12 gut group	0.09	0.11	0.09	0.19	0.22	0.30	0.38	0.45	0.30	0.34	0.37	0.71	0.25	0.39	0.29	0.33
Lentisphaerae; low abundant Lentisphaerae	0.01	0.00	0.00	0.00	0.01	0.00	0.00	0.00	0.01	0.01	0.00	0.01	0.00	0.01	0.01	0.01
Spirochaetes; Spirochaetes (class); Spirochaetales; Spirochaetaceae	1.63	2.16	2.13	3.93	4.32	6.21	6.00	6.17	3.23	5.20	4.51	3.12	3.16	2.72	2.11	3.31
Spirochaetes; Spirochaetes (class); Spirochaetales; PL-11B10	0.00	0.00	0.00	0.00	0.00	0.00	0.00	0.00	0.10	0.20	0.21	0.04	0.03	0.03	0.02	0.30
Spirochaetes; low abundant Spirochaetes	0.00	0.00	0.00	0.00	0.00	0.00	0.00	0.00	0.00	0.00	0.00	0.00	0.00	0.00	0.00	0.00
Candidate division TM7	0.02	0.02	0.02	0.01	0.15	0.10	0.11	0.10	0.05	0.05	0.05	0.07	0.09	0.07	0.07	0.04
Synergistetes; Synergista; Synergistales; Synergistaceae	0.06	0.09	0.05	0.05	0.03	0.06	0.02	0.02	0.06	0.06	0.06	0.02	0.03	0.06	0.02	0.03
Elusimicrobia; Elusimicrobia (class); Elusimicrobiales; Elusimicrobiaceae	0.01	0.00	0.00	0.01	0.19	0.21	0.21	0.17	0.08	0.08	0.13	0.23	0.20	0.16	0.12	0.14
Elusimicrobia; low abundant Elusimicrobia	0.01	0.00	0.01	0.01	0.00	0.00	0.00	0.00	0.01	0.01	0.01	0.01	0.00	0.00	0.01	0.01
Candidate division SR1	0.00	0.00	0.00	0.00	0.03	0.02	0.02	0.02	0.10	0.13	0.11	0.12	0.15	0.15	0.12	0.13
Fibrobacteres; Fibrobacteria; Fibrobacterales; Fibrobacteraceae	0.70	1.47	1.13	1.48	3.65	11.18	11.46	8.65	2.84	5.15	5.17	7.50	2.95	4.64	3.60	4.60
Fibrobacteres; low abundant Fibrobacteres	0.00	0.00	0.00	0.00	0.00	0.00	0.00	0.00	0.00	0.00	0.00	0.00	0.00	0.00	0.00	0.00
Candidate division TM6	0.01	0.02	0.02	0.03	0.00	0.00	0.00	0.00	0.01	0.01	0.02	0.03	0.02	0.01	0.02	0.04
low abundant Bacteria	0.01	0.02	0.02	0.02	0.02	0.01	0.02	0.02	0.03	0.04	0.02	0.03	0.02	0.02	0.02	0.03
Euryarchaeota; Thermoplasmata; Methanomassiliicoccales; Methanomassiliicoccales GIT clade	0.16	0.23	0.20	0.36	0.20	0.22	0.23	0.24	0.13	0.14	0.12	0.11	0.19	0.12	0.10	0.14
Euryarchaeota; Thermoplasmata; Thermoplasmatales; unassigned Thermoplasmatales	0.00	0.00	0.00	0.00	0.00	0.00	0.00	0.00	0.00	0.00	0.00	0.00	0.00	0.00	0.00	0.00
Euryarchaeota; Methanobacteria; Methanobacteriales; unassigned Methanobacteriaceae	0.03	0.03	0.02	0.06	0.02	0.01	0.01	0.01	0.03	0.03	0.03	0.02	0.01	0.02	0.01	0.03
Euryarchaeota; Methanobacteria; Methanobacteriales; Methanobrevibacter	0.10	0.08	0.06	0.17	0.19	0.13	0.10	0.17	0.09	0.10	0.12	0.18	0.21	0.15	0.09	0.11
Euryarchaeota; Methanobacteria; Methanobacteriales; Methanosphaera	0.01	0.01	0.01	0.01	0.02	0.02	0.01	0.01	0.00	0.00	0.01	0.01	0.01	0.01	0.00	0.02
Euryarchaeota; Methanomicrobia; Methanosarcinales; Methanosarcinaceae	0.00	0.00	0.00	0.00	0.00	0.00	0.00	0.00	0.00	0.00	0.00	0.00	0.00	0.00	0.00	0.00

[a] Taxa which could not be assigned on family level and/or showed relative abundance ≤ 0.01 % level are shown on higher taxonomic levels
