# Supplementary material for: Holistic Assessment of Rumen Microbiome Dynamics through Quantitative Metatranscriptomics Reveals Multifunctional Redundancy during Key Steps of Anaerobic Feed Degradation
Source: mSystems. 2018 Aug 7;3(4):e00038-18. doi: 10.1128/mSystems.00038-18 (PMC6081794; doi:10.1128/mSystems.00038-18)
Supplement: TABLE S5 [file sys004182253st5.pdf]

Supplementary Table S5. Significantly higher expressed functions one hour after ruminant feed intake.

Fig. 3	Differential gene expression analysis (edgeR)			SEED subsystems		
ID	**SEED functions** (level 4)	**logFC**	**FDR**[a]	**level 1**	**level 2**	**level 3**
1	Fumarate reductase subunit C	4.70	***	Respiration	Electron donating reactions	Succinate_dehydrogenase
2	Putative phosphatase YqaB	3.76	**	DNA Metabolism	DNA repair	2-phosphoglycolate_salvage
3	Lipid A biosynthesis (KDO) 2-(lauroyl)-lipid IVA acyltransferase	2.68	*	Cell Wall and Capsule	Gram-Negative cell wall components	KDO2-Lipid_A_biosynthesis
4	DNA-directed RNA polymerase subunit B (EC 2.7.7.6)	2.18	**	RNA Metabolism	Transcription	RNA_polymerase_archaeal
5	DNA-directed RNA polymerase subunit N (EC 2.7.7.6)	2.01	***	RNA Metabolism	Transcription	RNA_polymerase_archaeal
6	LSU ribosomal protein L7Ae	1.49	**	Protein Metabolism	Protein biosynthesis	Ribosome_LSU_bacterial
7	3-hydroxybutyryl-CoA dehydratase (EC 4.2.1.55)	1.34	*	Carbohydrates	Fermentation	Acetyl-CoA_fermentation_to_Butyrate
8	Phosphoenolpyruvate carboxykinase [ATP] (EC 4.1.1.49)	1.18	**	Carbohydrates	Central carbohydrate metabolism	Pyruvate_metabolism_I:_anaplerotic_reactions,_PEP
9	Na()-translocating NADH-quinone reductase subunit F	1.17	*	Respiration	Electron donating reactions	Na()-translocating_NADH-quinone_oxidoreductase[2]
10	LSU ribosomal protein L14p (L23e)	1.11	***	Protein Metabolism	Protein biosynthesis	Ribosome_LSU_bacterial
11	Na()-translocating NADH-quinone reductase subunit C	1.03	**	Respiration	Electron donating reactions	Na()-translocating_NADH-quinone_oxidoreductase[b]
12	NADH-ubiquinone oxidoreductase chain L (EC 1.6.5.3)	1.01	**	Respiration	Electron donating reactions	Respiratory_Complex_I
13	Choline binding protein A	1.00	**	Stress Response	Osmotic stress	Choline_and_Betaine_Uptake_and_Betaine_Biosynthesis
14	Cysteine desulfurase (EC 2.8.1.7), NifS subfamily	1.00	*	Amino Acids and Derivatives	Alanine, serine, and glycine	Alanine_biosynthesis
15	6-phospho-beta-glucosidase (EC 3.2.1.86)	0.92	*	Carbohydrates	Di- and oligosaccharides	Beta-Glucoside_Metabolism
16	LSU ribosomal protein L24p (L26e)	0.89	***	Protein Metabolism	Protein biosynthesis	Ribosome_LSU_bacterial
17	LSU ribosomal protein L10p (P0)	0.87	**	Protein Metabolism	Protein biosynthesis	Ribosome_LSU_bacterial
18	LSU ribosomal protein L31p	0.84	**	Protein Metabolism	Protein biosynthesis	Ribosome_LSU_bacterial
19	LSU ribosomal protein L27p	0.82	***	Protein Metabolism	Protein biosynthesis	Ribosome_LSU_bacterial
20	SSU ribosomal protein S8p (S15Ae)	0.80	***	Protein Metabolism	Protein biosynthesis	Ribosome_SSU_bacterial
21	SSU ribosomal protein S4p (S9e)	0.79	***	Protein Metabolism	Protein biosynthesis	Ribosome_SSU_bacterial
22	LSU ribosomal protein L18p (L5e)	0.78	**	Protein Metabolism	Protein biosynthesis	Ribosome_LSU_bacterial
23	SSU ribosomal protein S5p (S2e)	0.77	***	Protein Metabolism	Protein biosynthesis	Ribosome_SSU_bacterial
24	SSU ribosomal protein S3p (S3e)	0.76	***	Protein Metabolism	Protein biosynthesis	Ribosome_SSU_bacterial
25	SSU ribosomal protein S16p	0.75	**	Protein Metabolism	Protein biosynthesis	Ribosome_SSU_bacterial
26	Biosynthetic arginine decarboxylase (EC 4.1.1.19)	0.75	*	Stress Response	Acid stress	Acid_resistance_mechanisms
27	SSU ribosomal protein S11p (S14e)	0.73	**	Protein Metabolism	Protein biosynthesis	Ribosome_SSU_bacterial
28	Ketol-acid reductoisomerase (EC 1.1.1.86)	0.71	*	Amino Acids and Derivatives	Branched-chain amino acids	Branched-Chain_Amino_Acid_Biosynthesis
29	LSU ribosomal protein L17p	0.70	**	Protein Metabolism	Protein biosynthesis	Ribosome_LSU_bacterial
30	SSU ribosomal protein S13p (S18e)	0.69	**	Protein Metabolism	Protein biosynthesis	Ribosome_SSU_bacterial
31	Translation elongation factor G	0.68	**	Protein Metabolism	Protein biosynthesis	Universal_GTPases
32	SSU ribosomal protein S14p (S29e)	0.68	**	Protein Metabolism	Protein biosynthesis	Ribosome_SSU_bacterial
33	LSU ribosomal protein L11p (L12e)	0.68	*	Protein Metabolism	Protein biosynthesis	Ribosome_LSU_bacterial
34	LSU ribosomal protein L1p (L10Ae)	0.67	**	Protein Metabolism	Protein biosynthesis	Ribosome_LSU_bacterial
35	LSU ribosomal protein L3p (L3e)	0.67	**	Protein Metabolism	Protein biosynthesis	Ribosome_LSU_bacterial
36	LSU ribosomal protein L5p (L11e)	0.66	**	Protein Metabolism	Protein biosynthesis	Ribosome_LSU_bacterial
37	SSU ribosomal protein S18p	0.65	*	Protein Metabolism	Protein biosynthesis	Ribosome_SSU_bacterial
38	LSU ribosomal protein L30p (L7e)	0.65	*	Protein Metabolism	Protein biosynthesis	Ribosome_LSU_bacterial
39	LSU ribosomal protein L16p (L10e)	0.62	**	Protein Metabolism	Protein biosynthesis	Ribosome_LSU_bacterial
40	LSU ribosomal protein L33p	0.62	*	Protein Metabolism	Protein biosynthesis	Ribosome_LSU_bacterial
41	SSU ribosomal protein S1p	0.54	*	Protein Metabolism	Protein biosynthesis	Ribosome_SSU_bacterial
42	SSU ribosomal protein S17p (S11e)	0.52	*	Protein Metabolism	Protein biosynthesis	Ribosome_SSU_bacterial
43	SSU ribosomal protein S9p (S16e)	0.50	*	Protein Metabolism	Protein biosynthesis	Ribosome_SSU_bacterial

[a] FDR, corrected p-value, *** ≤ 0.001, ** ≤ 0.01, * ≤ 0.05; [b] Na()-translocating_NADH-quinone_oxidoreductase_and_rnf-like_group_of_electron_transport_complexes
