# Supplementary material for: Holistic Assessment of Rumen Microbiome Dynamics through Quantitative Metatranscriptomics Reveals Multifunctional Redundancy during Key Steps of Anaerobic Feed Degradation
Source: mSystems. 2018 Aug 7;3(4):e00038-18. doi: 10.1128/mSystems.00038-18 (PMC6081794; doi:10.1128/mSystems.00038-18)
Supplement: TABLE S6 [file sys004182253st6.pdf]

Supplementary Table S6. Raw count data of carbohydrate active enzymes detected in the rumen metatranscriptomes. c1 - c4, cow 1 - 4; 7h (t0), 8h (t1), 10h (t3), 12h (t5)

Categorie	pfam model	name	c1 7h	c2 7h	c3 7h	c4 7h	c1 8h	c2 8h	c3 8h	c4 8h	c1 10h	c2 10h	c3 10h	c4 10h	c1 12h	c2 12h	c3 12h	c4 12h
Lignin - phenolics	PF02578.10	Cu-oxidase_4	47	27	49	30	61	42	54	36	65	43	57	32	54	41	47	45
	PF00775.16	Dioxygenase_C	0	0	0	0	0	0	0	1	0	0	0	0	0	0	0	0
Cellulases	PF12876.2	Cellulase_like	1	1	2	1	1	6	0	0	1	4	2	0	0	3	1	1
	PF00150.13	Cellulase	1419	404	537	834	901	747	732	441	1239	473	387	1544	692	351	522	525
	PF01341.12	Glyco_hydro_6	3	1	2	1	2	3	1	2	4	1	4	0	8	2	2	5
	PF00759.14	Glyco_hydro_9	2370	546	655	1123	1232	1033	922	725	1641	644	562	2273	804	489	570	629
	PF02011.10	Glyco_hydro_48	68	276	125	208	55	267	209	183	58	372	242	223	119	338	212	236
	PF12891.2	Glyco_hydro_44	0	14	9	12	2	12	7	5	1	16	16	9	7	24	7	12
	PF02015.11	Glyco_hydro_45	2390	301	575	1224	1491	861	732	566	2096	246	392	2731	1174	151	560	439
Hemicellulases	PF01270.12	Glyco_hydro_8	14	39	48	63	16	52	41	32	17	68	49	51	41	75	100	74
	PF00331.15	Glyco_hydro_10	1197	450	522	593	968	517	557	373	1319	412	393	816	747	381	478	454
	PF00457.12	Glyco_hydro_11	395	113	120	216	274	129	143	128	297	99	88	367	201	123	147	146
	PF02156.10	Glyco_hydro_26	444	119	149	258	262	219	207	144	451	130	159	450	250	120	155	153
	PF00295.12	Glyco_hydro_28	79	35	56	38	84	34	54	29	93	35	46	37	79	35	51	53
	PF07745.8	Glyco_hydro_53	143	77	183	98	104	96	151	95	179	112	127	84	184	105	137	154
Pectinases	PF00295	Glyco_hydro_28	79	35	56	38	84	34	54	29	93	35	46	37	79	35	51	53
Chitinases	PF00704	Glyco_hydro_18	111	9	26	23	49	18	25	7	82	15	27	43	110	23	24	26
Glucosylceramidase	PF02055	Glyco_hydro_30	100	17	29	52	63	16	18	14	70	8	10	78	44	10	25	18
	PF06964.7	Alpha-L-AF_C	178	78	190	88	163	122	144	85	236	73	103	77	165	110	131	125
	PF09206.6	ArabFuran-catal	2	6	2	2	0	4	2	1	0	5	3	1	3	7	5	1
	PF07477.7	Glyco_hydro_67C	48	32	29	27	41	27	33	41	57	11	33	26	65	24	42	22
	PF07488.7	Glyco_hydro_67M	74	22	57	31	55	34	54	27	97	14	46	27	70	23	55	51
	PF03648.9	Glyco_hydro_67N	2	1	3	0	3	2	1	0	3	3	3	1	3	0	2	3
	PF05592.6	Bac_rhamnosid	127	41	104	54	85	55	98	49	124	55	77	89	103	53	81	79
	PF08531.5	Bac_rhamnosid_N	45	28	38	16	28	14	31	19	46	22	17	22	38	26	33	22
Starch degrading enzymes	PF00128	Alpha-amylase	792	390	477	492	696	519	665	515	1097	524	674	882	1167	543	596	702
	PF03065	Glyco_hydro_57	221	77	156	69	123	83	122	85	275	86	112	85	233	88	119	133
	PF02446	Glyco_hydro_77	651	413	513	549	651	802	975	497	1307	888	817	831	1075	717	423	650
Mannan, Galactomannan degrading	PF02156	Glyco_hydro_26	444	119	149	258	262	219	207	144	451	130	159	450	250	120	155	153
	PF03663	Glyco_hydro_76	10	3	6	0	7	1	0	2	7	1	4	5	6	2	6	5
Oligosaccharide hydrolases	PF00232.13	Glyco_hydro_1	58	56	78	51	98	98	74	46	95	55	42	47	158	83	88	81
	PF00703.16	Glyco_hydro_2	96	43	94	56	49	53	60	41	110	46	65	72	82	61	91	60
	PF02836.12	Glyco_hydro_2_C	327	163	252	169	225	205	261	175	368	186	223	227	316	235	308	248
	PF02837.13	Glyco_hydro_2_N	298	158	284	183	204	193	264	148	351	194	201	195	309	211	294	227
	PF00933.16	Glyco_hydro_3	644	440	559	414	583	382	564	509	717	393	470	828	758	483	721	491
	PF01915.17	Glyco_hydro_3_C	343	184	305	215	325	205	297	240	418	200	186	403	412	226	381	256
	PF01120.12	Alpha_L_fucos	86	39	74	41	58	40	65	49	68	36	50	35	78	66	71	67
	PF01301.14	Glyco_hydro_35	73	29	66	35	45	39	67	56	75	36	58	39	67	42	60	60
	PF01074.17	Glyco_hydro_38	9	6	12	9	8	12	9	6	17	5	8	8	8	8	15	14

Categorie	pfam model	name	c1 7h	c2 7h	c3 7h	c4 7h	c1 8h	c2 8h	c3 8h	c4 8h	c1 10h	c2 10h	c3 10h	c4 10h	c1 12h	c2 12h	c3 12h	c4 12h
Oligosaccharide hydrolases	PF07748.8	Glyco_hydro_38_C	2	4	6	1	3	2	0	3	5	3	2	0	2	1	5	4
	PF01229.12	Glyco_hydro_39	4	3	6	3	4	3	6	0	2	4	1	5	3	4	6	7
	PF02449.10	Glyco_hydro_42	9	12	14	14	10	16	17	15	8	15	17	3	14	14	18	15
	PF08533.5	Glyco_hydro_42_C	0	3	0	0	0	2	0	0	0	1	1	2	0	1	2	2
	PF04616.9	Glyco_hydro_43	749	265	486	410	658	342	444	278	845	273	363	487	674	331	516	407
	PF07470	Glyco_hydro_88	236	70	228	108	183	109	175	116	280	95	128	143	213	100	170	132
	PF03632	Glyco_hydro_65m	8	3	9	4	3	3	8	4	2	5	15	5	4	6	4	6
	PF02065	Melibiase	158	98	138	105	211	131	135	105	193	96	95	125	146	136	116	111
	PF01204	Trehalase	2	5	2	4	0	3	5	0	1	1	1	1	4	3	6	4
Other glycoside hydrolases	PF02056	Glyco_hydro_4	6	5	2	9	13	10	4	4	6	5	10	5	9	5	10	6
	PF00722	Glyco_hydro_16	129	50	58	52	121	85	66	57	93	57	55	78	187	75	101	91
	PF00728	Glyco_hydro_20	88	29	42	33	46	22	42	34	56	22	46	46	49	28	56	52
	PF01055	Glyco_hydro_31	465	172	347	241	428	248	339	251	615	186	268	341	503	196	317	284
	PF00251	Glyco_hydro_32N	374	133	197	167	212	161	182	289	185	90	149	567	217	94	391	203
	PF02012	BNR	2	5	0	2	0	5	2	0	0	5	1	1	1	0	2	1
	PF03200	Glyco_hydro_63	0	0	0	2	1	0	0	1	1	1	3	3	1	1	0	3
	PF03639	Glyco_hydro_81	0	0	1	0	1	1	1	1	2	0	1	0	1	1	1	2
	PF05089	NAGLU	37	55	33	37	39	37	43	38	37	15	46	22	38	37	33	46
	PF03562	MltA	0	1	0	0	0	0	0	1	1	2	1	2	0	0	2	3
Lysozymes	PF01183	Glyco_hydro_25	187	112	186	171	178	180	142	131	223	113	151	361	245	125	182	196
Cellulose binding	PF00553	CBM_2	2	0	2	1	1	1	3	1	1	3	3	2	4	2	1	1
	PF00942	CBM_3	31	5	9	15	15	14	6	5	25	4	2	19	10	11	6	9
	PF02018	CBM_4_9	76	100	71	78	64	112	80	51	91	118	60	99	89	120	104	93
	PF03425	CBM_11	1	19	6	10	4	9	10	13	2	38	18	18	7	33	24	11
Chitin binding	PF02839	CBM_5_12	40	0	18	16	18	7	21	16	43	6	13	39	20	2	2	14
Starch binding	PF00686	CBM_20	96	20	58	41	66	67	81	40	174	74	64	60	142	55	42	65
	PF02903	Alpha-amylase_N	3	5	1	8	19	8	9	8	18	14	7	3	10	6	4	5
	PF03423	CBM_25	0	0	3	0	0	1	0	0	0	1	0	0	0	2	1	1
Oligosaccaride binding	PF03422	CBM_6	212	164	143	170	164	171	145	128	219	201	147	218	176	207	206	173
Other carbohydrate binding modules (CBM)	PF00652	Ricin_B_lectin	24	11	5	11	10	15	3	5	8	12	5	18	7	12	13	6
	PF00754	F5_F8_type_C	35	24	37	20	27	12	19	11	37	21	12	19	30	30	17	30
	PF08305	NPCBM	3	4	6	12	3	10	5	2	8	6	9	9	3	9	11	8
Bacterial cell wall degradation (CBM)	PF01476	LysM	213	138	257	202	213	176	249	169	283	222	301	181	261	259	253	333
